# Single-nucleus RNA sequencing and deep tissue proteomics reveal distinct tumour microenvironment in stage-I and II cervical cancer

**DOI:** 10.1186/s13046-023-02598-0

**Published:** 2023-01-23

**Authors:** Xiaosong Liu, Guoying Ni, Pingping Zhang, Hejie Li, Junjie Li, Bernardo Cavallazzi Sebold, Xiaolian Wu, Guoqiang Chen, Songhua Yuan, Tianfang Wang

**Affiliations:** 1 Cancer Research Institute, First People’s Hospital of Foshan, Foshan, 528000 Guangdong China; 2 The First Affiliated Hospital/School of Clinical Medicineof, Guangdong Pharmaceutical University, Guangzhou, 510080 Guangdong China; 3 Centre for Bioinnovation, University of the Sunshine Coast, Maroochydore BC, QLD 4558 Australia; 4 School of Science, Technology and Engineering, University of the Sunshine Coast, Maroochydore BC, QLD 4558 Australia; 5 Department of Gynaecology, First People’s Hospital of Foshan, Foshan, 528000 Guangdong China

**Keywords:** Single-nucleus RNA sequencing, Quantitative proteomics, Tumour microenvironment, Cervical cancer, Macrophage, Collagen

## Abstract

**Background:**

Cervical cancer (CC) is the 3^rd^ most common cancer in women and the 4^th^ leading cause of deaths in gynaecological malignancies, yet the exact progression of CC is inconclusive, mainly due to the high complexity of the changing tumour microenvironment (TME) at different stages of tumorigenesis. Importantly, a detailed comparative single-nucleus transcriptomic analysis of tumour microenvironment (TME) of CC patients at different stages is lacking.

**Methods:**

In this study, a total of 42,928 and 29,200 nuclei isolated from the tumour tissues of stage-I and II CC patients and subjected to single-nucleus RNA sequencing (snRNA-seq) analysis. The cell heterogeneity and functions were comparatively investigated using bioinformatic tools. In addition, label-free quantitative mass spectrometry based proteomic analysis was carried out. The proteome profiles of stage-I and II CC patients were compared, and an integrative analysis with the snRNA-seq was performed.

**Results:**

Compared with the stage-I CC (CCI) patients, the immune response relevant signalling pathways were largely suppressed in various immune cells of the stage-II CC (CCII) patients, yet the signalling associated with cell and tissue development was enriched, as well as metabolism for energy production suggested by the upregulation of genes associated with mitochondria. This was consistent with the quantitative proteomic analysis that showed the dominance of proteins promoting cell growth and intercellular matrix development in the TME of CCII group. The interferon-α and γ responses appeared the most activated pathways in many cell populations of the CCI patients. Several collagens, such as *COL12A1*, *COL5A1*, *COL4A1* and *COL4A2*, were found significantly upregulated in the CCII group, suggesting their roles in diagnosing CC progression. A novel transcript *AC244205.1* was detected as the most upregulated gene in CCII patients, and its possible mechanistic role in CC may be investigated further.

**Conclusions:**

Our study provides important resources for decoding the progression of CC and set the foundation for developing novel approaches for diagnosing CC and tackling the immunosuppressive TME.

**Supplementary Information:**

The online version contains supplementary material available at 10.1186/s13046-023-02598-0.

## Background

Cervical cancers (CC) are the 3^rd^ most common cancer in women worldwide [1], and human papillomavirus (HPV)16 and 18 account for more than 70% of CC [2]. Clinically, the International Federation of Gynecology and Obstetrics (FIGO) cervical cancer staging system is the most powerful prognostic factor in patients with cervical cancer and useful guidance for treatment [3, 4]. Stage-I CC cells have grown from the surface of the cervix into deeper tissues, while stage-II cancer is 4 cm or larger, and grows beyond the cervix and uterus, but hasn’t spread to the walls of the pelvis or the lower part of the vagina. Surgical, radio- and chemotherapy or a combination of these therapies are used for the treatment of CC [5]. Immune checkpoint inhibitor therapy has been recommended as a second-line treatment for PD-L1 positive or MSI-h/dMMR tumours [6]. However, the immunopathological profiles of these two stages have not been studied clearly, leading to the difficulty in the selection of stage-specific immunotherapy strategy.

Research over the last two decades has demonstrated that both innate and adaptive immune systems participate in the elimination, equilibrium, and escape stages of the immune editing process [7, 8]. Immune surveillance and subsequent immune editing theory suggest that the immune system can either positively or negatively influence tumour development. Reduced immune recognition, increased tumour cell survival, or development of an immunosuppressive tumour microenvironment (TME) contribute to the tumour escape stage [9]. The TME establishment is a slow process, and recent studies showed that the early- and late-stage TMEs of multiple cancers have cells with different cell heterogeneity and functions [10–14]. Recent advances in multi-omics and single-cell RNA sequencing (scRNA-seq) techniques have contributed significantly to the characterisation of TME, which have resulted in the discovery of more cell types and their response to therapies [15–18]. As an example, in a TC-1 tumour model, six tumour-associated macrophage populations with distinct genomic signatures were present in the TME, reflecting a complex macrophage development, compared with the traditional M1 and M2 paradigm [19, 20].

In terms of the TME of CC, a recent scRNA-seq analysis has found the enrichment of PI3K/AKT pathway supported by differentially expressed genes between chemoresistant and chemosensitive patients [17]. The mutation in *NFKB1* (G430E) was shown to significantly increase mutant allele frequency after radiotherapy, indicating its role as a potential molecular target in CC radiation therapy [21]. Another study compared the CC and the adjacent normal tissues by scRNA-seq analysis, which discovered a subset of cancer stem cells (CSCs) significantly related to tumour progression; in addition, metabolism-related signalling pathway was enhanced in the endothelial cells of the TME, associated with upregulation of *TAGLN2*, *KLF5*, *STAT1*, and *STAT2 *[15]. Besides, the immune cells and mesenchymal cells of the normal cervix, intraepithelial neoplasia, primary tumour, and metastatic lymph node tissues were comparatively investigated using scRNA-seq, which identified a low and late activated TME in intraepithelial neoplasia, whereas metastatic lymph node showed early activated immune response [22]. However, the efforts in profiling all cell types of the TME of different stage CC to understand their cellular biology are still limited.

In this study, we comprehensively compared the cell heterogeneity in the TME of stage-I and II CC patients. We performed high-precision single-nucleus RNA sequencing (snRNA-seq) analysis with the surgical tissues isolated from four stage-I and three stage-II CC patients, respectively. We detected, on average, 1,900 genes at a depth of ~ 250,000 reads per nucleus in about 80,000 tumour cells. The tumour cells were clustered into different types according to their transcriptome profiles, and the subtypes of selected immune cells were identified. We then employed label-free quantitative proteomic analysis to compare the overall proteome profiles of the tumour tissues, and their correlation with snRNA-seq analysis was revealed to address the basic questions in cell type and function raised above. We identified the distinct phenotypes in the TMEs of the two stages, and the marker genes specific to stage-II CC included many collagens and a novel transcript *AC244205.1*. Survival analysis based on The Cancer Genome Atlas data further supported the correlation between the collagen expression and CC patient survival, suggesting that they can serve as candidate targets for tumour therapy of late-stage CC patients. Our work provides novel insights into the molecular characteristics of the progression of CC.

## Methods

### Patients

Pathological confirmed tumour samples of cervical cancer patients without chemo-, radiotherapy, HBV, EBV and HIV negative underwent surgical operation were collected and stored in liquid nitrogen till use. The patient information is summarised in Table S1. The human ethnics number for conducting the current research is L2016.

### Isolation of tumours and single-nucleus transcriptomics

Freshly obtained CC tissues were immediately processed for nucleus isolation, sequencing, and library preparation, according to the guidelines of 10 × Genomics (10X Genomics, USA). Approximately 500 mg of tumour tissue from each patient was dissociated into a single-nucleus suspension. The tumour tissue was homogenised in ice-cold homogenisation buffer (0.25 M sucrose, 5 mM CaCl_2_, 3 mM MgAc2, 10 mM Tris–HCl (pH = 8.0), 1 mM DTT, 0.1 mM EDTA, 1 × protease inhibitor (Thermo Scientific, cat no. 78425), and 1 U/μL RiboLock RNase Inhibitor (Thermo Scientific, cat no. O0381)) with pestle strokes. Next, the homogenates were filtered through a 70 μm cell strainer to collect the nuclear fraction in to 15 ml Falcon tube, with a volume about 1 ml. The nuclear fraction was mixed with an equal volume of 50% iodixanol solution (0.16 M sucrose, 1 0 mM NaCl_2_, 3 mM MgCl_2_, 10 mM Tris HCl (pH 7.4), 1 U/μl RiboLock R Nase Inhibitor, 1 mM DTT, 0.1 mM PMSF Protease Inhibitor (Thermo Scientific, cat no. 36978)), to a final concentration of 25%, then add 1 mL 33% iodixanol solution to the bottom of the tube, followed by adding on top of a 30% iodixanol solution. This solution was mixed by inverting for 10 times and then centrifuged for 8 min at 500 × g at 4 °C. After the myelin layer was removed from the top of the gradient, the nuclei were collected from the 30% iodixanol interface. The nuclei were resuspended in nuclear wash buffer and resuspension buffer (0.04% bovine serum albumin, 0.2 U/μL RiboLock RNase inhibitor, 500 mM mannitol and 0.1 mM PMSF protease inhibitor in PBS) and pelleted for 5 min at 500 × g and 4 °C. The nuclei were filtered through a 40 μm cell strainer to remove cell debris and large clumps. The nuclear concentration was assessed using trypan blue counterstaining by a Bio-rad TC20. Finally, the nuclear concentration was adjusted to 700–1200 nuclei/μl, and the nuclei were examined with a 10X Chromium platform. Reverse transcription, cDNA amplification and library preparation were performed based on the protocol from the manufacturer.

Raw reads were preprocessed using Cell Ranger (version 3.1.0) with the default parameters and aligned to the pre-mRNA reference (Ensemble_release 108.38, *Homo sapiens*). For quality control, cells with UMI counts < 8,000 or a percentage of mitochondrial genes < 10%, and gene counts between 500 and 4,000 per nucleus were retained. Then, the global-scaling normalisation method “LogNormalise” was used to normalise the gene expression measurements for each cell by total expression, multiplied this by a scale factor (10,000 by default), and log-transformed the result with the following formula. Seurat [23] was used to minimise the effects of batch effect and behavioural conditions, which identified 2,000 highly variable genes in each sample based on a variance stabilising transformation, to generate an integrated expression matrix.

$$\mathrm{gene \ expression \ level}\hspace{0.17em}=\hspace{0.17em}\mathrm{log}10\left(1 + \frac{{UMI}_{A}}{{UMI}_{Total}} \hspace{0.17em}\times \hspace{0.17em}10000\right)$$

After data integration and scaling, principal component analysis (PCA) was used dimensional reduction, and appropriate principal components were selected for clustering and subsequent analysis. The detailed method for clustering cells based on gene expression was described in detail elsewhere [19, 20]. In brief, a shared-nearest neighbour (SNN) graph was used to draw edges around cells with similar gene expression based on the euclidean distance in PCA space. The edge weights were refined between any two cells according to their Jaccard distance. The modularity optimisation techniques were applied to iteratively group cells together [24], for the purpose of optimising the standard modularity function.

### Differentially expressed gene (DEP)

Expression value of each gene in given cluster were compared against the rest of cells using Wilcoxon rank sum test. Significant upregulated genes were identified using three criteria: (i) the expression of the genes ≥ 1.28-fold in the target cluster relative to other clusters; (ii) the genes were expressed in more than 25% of the cells of the target cluster; and (iii) *P*-value is < 0.05.

### Cell cycle analysis

The Seurat R package was used to assign a cell cycle score to each cell based on the 100 marker genes for G1/S phase, 113 marker genes for S phase, 133 marker genes for G2/M phase, 151 marker genes for M phase and 106 marker genes for M/G1 phase, respectively [25]. Cells with the highest score less than 0.3 was identified as non-cycling cells [26].

### Protein extraction and trypsin digestion

Tumour tissue samples were transferred into lysis buffer (2% SDS, 7 M urea, 1 mg/mL protease inhibitor cocktail), and homogenised for 3 min on ice using an ultrasonic homogeniser (Sonics & Materials Inc VCX130). The homogenate was centrifuged at 15,000 rpm for 15 min at 4℃, and the supernatant was collected. BCA Protein Assay Kit (ThermoFisher Scientific, MA, US) was used to determine the protein concentration of the supernatant. A total of 50 μg protein extracted from each sample was suspended in 50 μL solution, reduced by adding 1 μL 1 M dithiotreitolat 55 °C for 1 h, alkylated by adding 5 μL 20 mM iodoacetamide in the dark at 37 °C for 1 h. Then the sample was precipitated using 300 μL prechilled acetone at -20 ℃ overnight. The precipitate was washed twice with cold acetone and then resuspended in 50 mM ammonium bicarbonate, followed by digestion with sequence-grade modified trypsin (Promega, Madison, WI) at a substrate/enzyme ratio of 50:1 (w/w) at 37 °C for 16 h.

### High pH reverse phase separation

The peptide mixture was resuspended in the buffer A (buffer A: 20 mM ammonium formate in water, pH 10.0, adjusted with ammonium hydroxide), and then fractionated by high pH separation using Ultimate 3000 system (ThermoFisher Scientific, MA, US) connected to a reverse phase column (XBridge C18 column, 4.6 mm × 250 mm, 5 μm (Waters Corporation, MA, USA). High pH separation was performed using a linear gradient, starting from 5% B to 45% B in 40 min (B: 20 mM ammonium formate in 80% acetonitrile (ACN), pH 10.0, adjusted with ammonium hydroxide). The column was re-equilibrated at the initial condition for 15 min. The column flow rate was maintained at 1 mL/min and the column temperature was maintained at 30℃. Twelve fractions were collected and lyophilised.

### nano-HPLC–MS/MS analysis

The peptides were re-dissolved in 30 μL solvent A (A: 0.1% formic acid in water) and analysed by online nanospray LC–MS/MS on an Orbitrap Fusion Lumos coupled to EASY-nLC 1200 system (Thermo Fisher Scientific, MA, US). Briefly, 3μL peptide sample was loaded onto the analytical column (Acclaim PepMapC18, 75 μm × 25 cm) with a 120-min gradient, from 5 to 35% B (B: 0.1% formic acid in ACN). The column flow rate was maintained at 200 nL/min with a column temperature of 40 °C. The electrospray voltage of 2 kV versus the inlet of the mass spectrometer was used. The mass spectrometer was run under data-independent acquisition (DIA) mode, and automatically switched between MS and MS/MS mode. The parameters were: (1) MS: scan range (m/z) = 350–1200, resolution = 120,000, AGC target = 1E6 and maximum injection time = 50 ms; (2) HCD-MS/MS: resolution = 30,000, AGC target = 1E6, collision energy = 32 and stepped CE = 5%; (3) DIA was performed with variable isolation window, and each window overlapped 1 m/z, and the window number was set to 60.

### Protein identification and quantification

Raw data of DIA were processed and analysed by Spectronaut X (Biognosys AG, Switzerland) with default parameters. The protein database derived from *Homo sapiens* genome was downloaded from NCBI (March 2021). Retention time prediction type was set to dynamic iRT. Data extraction was determined based on the extensive mass calibration. Q-value (FDR) cutoff on precursor and protein level was applied at 1%. Decoy generation was set to mutate, scrambled with a random number of AA position swamps (min = 2, max = length/2). All selected precursors passing the filters were used for quantification. The average top 3 filtered peptides were used to calculate the major group quantities. Normalisation was performed on averaging the abundance of all peptides. Medians were used for averaging. After Student’s *t*-test, differently expressed proteins (DEPs) were filtered if their *Q-value* < 0.05 and absolute AVG log2 ratio > 0.58. Principal component analysis (PCA) and correlation analysis were performed with R package gmodels. The correlation coefficient between two replicas was calculated to evaluate repeatability between samples.

### The protein domain and transcription factor (TF) analysis

The prediction of the protein domain used the Pfam_scan program [27]. The protein sequence was compared with the Pfam database to obtain the relevant annotation information of protein structure. The predicted protein sequences were compared by hmmscan with the TF database (animalTFDB [28]).

### Protein–protein interaction (PPI) analysis

Interactions among significantly regulated proteins were predicted using STRING [29]. All resources were selected to generate the network and ‘confidence’ was used as the meaning of network edges and the required interaction score of 0.700 was selected for all PPI, to highlight the most confident interactions. Neither the 1^st^ nor 2^nd^ shell of the PPI was included in this study. Protein without any interaction with other proteins was excluded from displaying in the network.

### Functional annotation and enrichment analysis

DEGs and DEPs were annotated against GO, KEGG and COG/KOG database to obtain their functions. Significant GO functions and pathways were examined within differentially expressed proteins with Q-value < 0.05. The enrichment of the pathways was analysed by Gene Set Enrichment Analysis (GSEA) with *P-*value < 0.05 using GSEA v4.1.0 [30].

### Survival analysis

The survival analyses were performed by the Cox proportional hazard model provided that the proportional hazard assumption was met based on weighted residuals using TIMER2.0 [31]. Hazard ratio was estimated relative to the lowest-risk group and assessed by a two-sided Wald test, *P*-value < 0.05 was significant.

## Results

### Identification of the tumour cell composition of stage-I and II cervical cancer

**Fig. 1:**
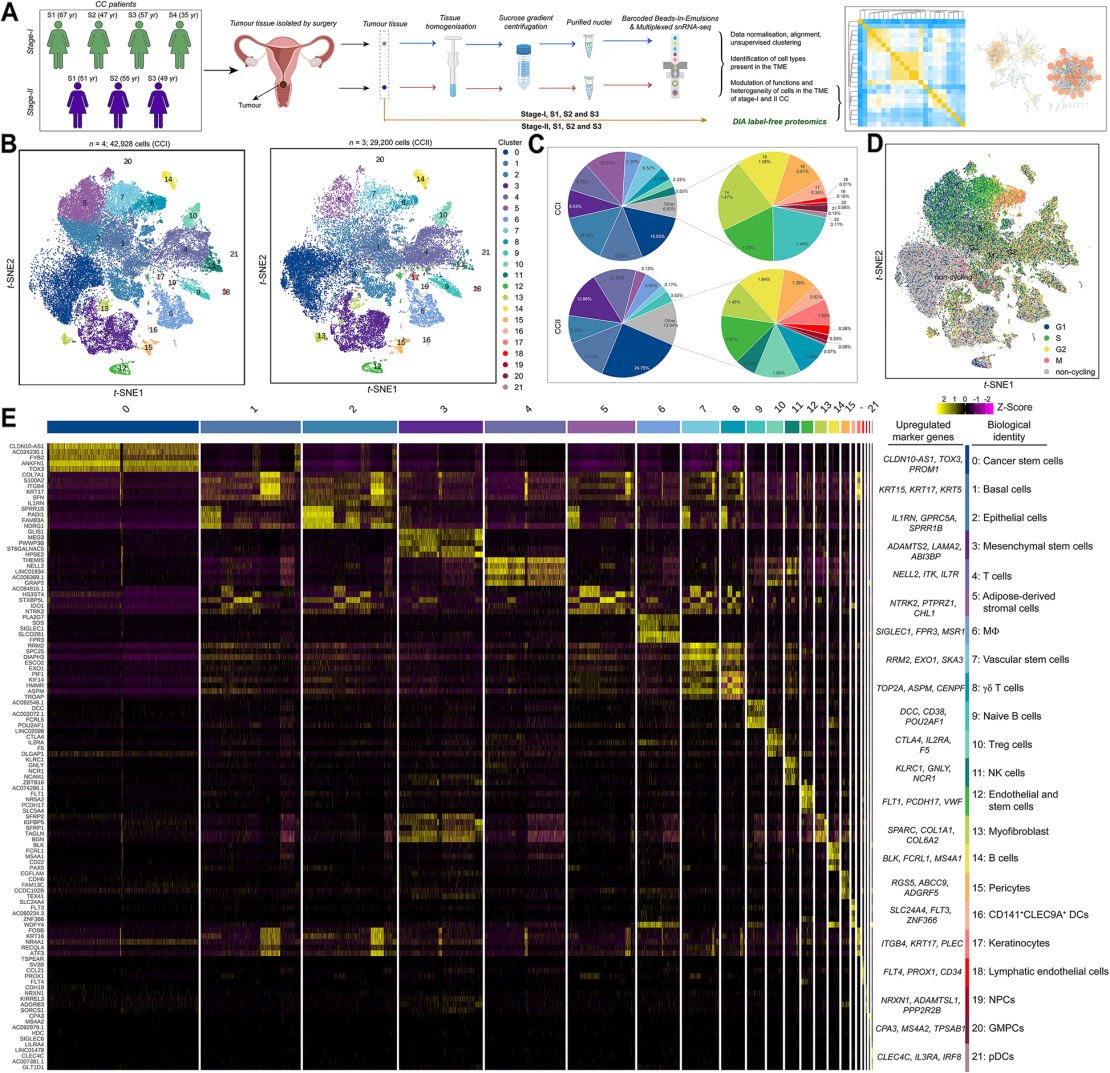
The snRNA-seq analysis of the tumour tissues of patients with stage-I and II cervical cancer. (**A**) Experimental design: the tumour tissues were collected from the patients at The First Affiliated Hospital/School of Clinical Medicine of Guangdong Pharmaceutical University. (**B**) t-Stochastic neighbour embedding (t-SNE) representation of aligned gene expression data in single nuclei extracted from the TME of CCI and CCII patients shows partition into 22 distinct clusters. (**C**) The proportions of the 22 cell clusters in the CCI and CCII groups. (**D**) The distributions of cell cycle phases in the t-SNE space. (**E**) Selected enriched genes used for biological identification of each cluster and the top 5 DEGs of each cluster (scale: log-transformed gene expression). MΦ represents macrophage; Treg cell, regulatory T cell; NK cell, natural killer cell; NPC, neural progenitor cell; GMPC, granulocyte-monocyte progenitor cell; pDC, plasmacytoid dendritic cell (see Table S2 for the full list of all marker genes detected)

We performed snRNA-seq experiments using all nuclei isolated from the tumour tissues of stage-I (*n* = 4) and II (*n* = 3) stage donors, respectively (Fig. 1A). After quality control, a total of 72,128 nuclei (42,928 stage-I and 29,200 stage-II) were used for downstream analysis (Fig. 1B and Fig. S1). Unsupervised clustering analysis revealed 22 cell types, which were present in both the stage-I (CCI) and II (CCII) groups, indicating that the cell-type identity was not strongly confounded by the ageing and stage effects (Fig. 1B; Figs. S2A and 2B). However, these cell types were not detected in all individual patients (Fig. S2C). The proportions of different cell types at the two stages were compared (Fig. 1C), and the population comparison among different samples were displayed in Figure S2D. Overall, the percentage populations of the cluster 3, 4, 9, 14, 15, 16, 17, 18, 19 were largely increased in the CCII group, whereas reduced proportions were observed for the cluster 2, 5, 7 and 20 (Fig. 1C and Table S1). Many of cluster 0 and 3 cells did not show the differential expression of the marker genes for definitive cell-cycle (non-cycling) (Fig. 1D). Cluster 1, 2 and 5 possessed high populations of cells at S or G1 cell cycle, while S and M cell cycles were more represented in cluster 7 and 8, respectively (Figs. S3A and 3B). CCI-4, CCII-2 and CCII-3 showed high proportion of non-cycling cells, while the other three CCI samples possessed more cells at G1 and S cell cycles (Figs. S3C and 3D).

The differentially expressed genes (DEGs) were analysed to determine cell type-specific marker genes (Fig. 1E and Table S2). The clusters were annotated with predicted cell-type identities based on known marker genes derived from the expert annotation in literature [32]. Correlation analysis of the 22 clusters showed that cluster 17 was least correlated with the other clusters, followed by cluster 13, implying distinct phenotypes present in these two clusters (Fig. S4). The cell types directly associated with immune response included T cells (cluster 4; marker genes: NELL2, ITK, and IL7R), macrophage (MΦ, cluster 6; SIGLEC1, FPR3, and MSR1) [33], γδT cells (cluster 8; TOP2A, ASPM, and CENPF), naïve B cells (cluster 9; DCC, CD38, POU2AF1) [34], regulatory T cells (Treg, cluster 10; CTLA4, IL2RA, and F5) [35], NK cells (cluster 11; KLRC1, GNLY, and NCR1), mature B cells (cluster 14; BLK, FCRL1, MS4A1) [36], CD141+CLEC9A+ DC (cluster 16; SLC24A4, FLT3, and ZNF366) [37], and plasmacytoid dendritic cells (pDCs, cluster 21; CLEC4C, IL3RA, and IRF8) [36, 38]. Several clusters had the features of stem cells, including cancer stem cells (CSCs) (cluster0; CLDN10-AS1, TOX3, and PROM1), mesenchymal stem cells (MSCs) (cluster 3; ADAMTS2, LAMA2, and ABI3BP), vascular stem cells (VSCs) (cluster 7; RRM2, EXO1, and SKA3), and endothelial cell/submandibular gland stem cells (cluster 12; FLT1, PCDH17, and VWF) [39]. Two clusters were annotated as progenitor cells, i.e., neural progenitor cells (NPCs, cluster 19; NRXN1, ADAMTSL1, and PPP2R2B) [40, 41] and granulocyte-monocyte progenitor cells (GMPCs, cluster 20; CPA3, MS4A2, and TPSAB1) [42]. Cluster 1 was characterised as basal cells, with significantly high expression of KRT15, KRT17, and KRT5. Epithelial cells and lymphatic endothelial cells were mainly detected in cluster 2 (IL1RN, GPRC5A, SPRR1B) and 18 (FLT4, PROX1, and CD34) [43], respectively. In addition, adipose-derived stromal cells (cluster 5; NTRK2, PTPRZ1, and CHL1), myofibroblast (cluster 13; SPARC, COL1A1, and COL6A2) [44], pericytes (cluster 15; RGS5, ABCC9, and ADGRF5), and astrocytes (cluster 17; FOSB, ATF3, and ITGB4) [45] were also present.

### Immunosuppressive and tumour-growth-promoting phenotypes dominated the macrophages in the TME of CCII patients

**Fig. 2:**
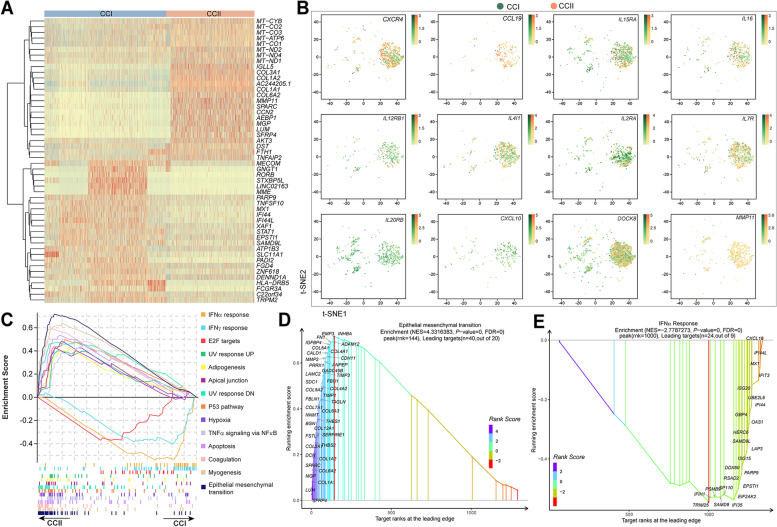
The MΦs of the CCI and CCII groups shows distinct levels of immune response. (**A**) The hierarchy diagram compares the expression and correlation of the top 50 DEGs between the CCI and CCII groups. (**B**) The 2D t-SNE graph compares the distribution of MΦs expressing (the normalised expression) the genes (FC > 1.2, *P*-value < 0.05) associated with the signalling of chemokines, cytokines and interleukins in the CCI and CCII groups. (**C**) The GSEA analysis of the Hallmark pathways enriched respectively by the DEGs of the CCI and CCII groups. The ranking of the genes significantly associated with the epithelial mesenchymal transition (**D**) and the IFN-α response pathway (**E**)

Relatively high populations of MΦs were identified in CCI (5.30%) and CCII (5.65%) patients, respectively. The gene expression analysis revealed 122 upregulated and 102 downregulated DEGs in the CCII groups relative to the CCI, with the log-transformed expression of the top 50 DEGs hierarchically compared in Fig. 2A (see Table S3 for the full list of DEGs and annotations). The expression of MΦ marker genes was comparatively downregulated in the CCII group, for instance, *C1QB*, *C1QC*, *STAT1* and *IFI44L*. In addition, many of chemokines, cytokines and interleukins exhibited significantly higher expression in the CCI group, including the signatures of M1-like MΦs, such as *IL12RB1*, *IL2RA* and *IL20RB* (Fig. 2B). The genes (*CCL19* and *MMP11*) that correlated with immune suppressive MΦs were elevated in the CCII group. The canonical pathways were analysed based on the respective transcriptome profiles using GSEA. It was evident that the epithelial-mesenchymal transition (EMT) was the most enriched pathway in the CCII group, supported by the upregulation of collagen family members, such as *COL1A1*, *COL1A2*, *COL6A2* and *COL3A1*. Furthermore, these collagens might function collaboratively with extracellular matrix synthesis regulating genes (e.g., *SFRP4*, *LUM*, *SPARC*, and *DCN*), to enhance tissue growth (Fig. 2C and 2D; Table S3). Besides, the enrichment of ‘P53 pathway’, ‘TNFα signalling via NFκB’, and ‘apoptosis’ was detected in the CCII group, and so were several other pathways closely related to the development of cell cytoskeleton. The elevation of genes encoding interferon-induced proteins in the CCI group strongly supported the activation of IFN-α and IFN-β response pathways, including *IFI44L*, *IFIT3*, *IFI44*, *IFI35*, and *IFIH1* (Fig. 2E).

**Fig. 3:**
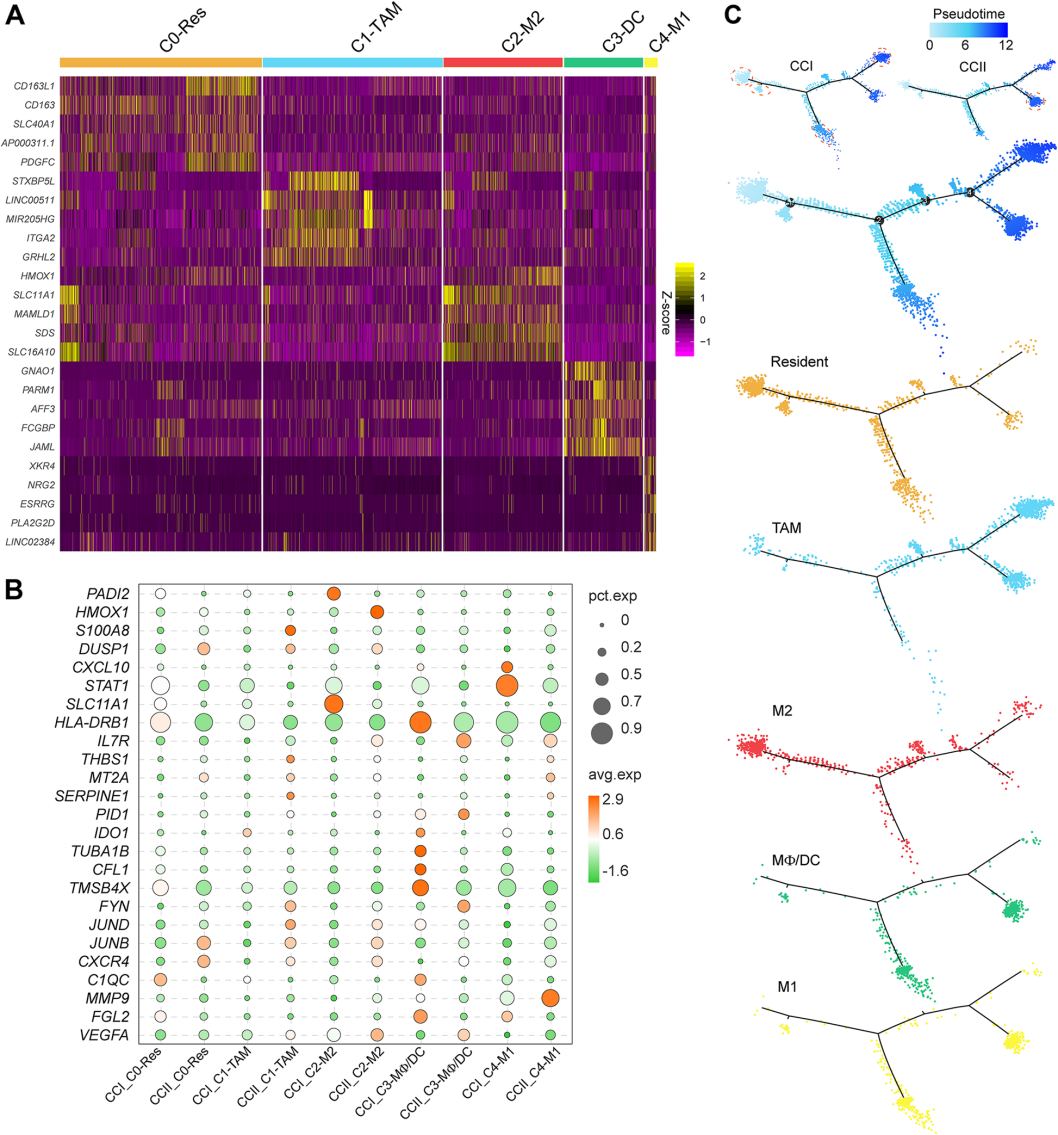
The analysis of macrophage heterogeneity of the CCI and CCII groups. (**A**) The subtype analysis of the macrophages. Five subpopulations were identified, including resident-like (C0-Res), tumour-associated (C1-TAM), M2-like (C2-M2), MΦ/DC (C3-DC) and M1-like (C4-M1). The expression of top 5 DEGs was compared across different subtypes. (**B**) Comparison of the average expression of selected DEGs associated with macrophage function. (**C**) Pseudotime trajectory analysis of macrophage subtypes

The MΦ subtypes were analysed to unveil the change in cell heterogeneity between the two stages. There were five subtypes (i.e., cluster-0 to 4) identified, with the expression of the top five marker genes compared in Fig. 3A. Cluster-0 had the phenotype of resident-like macrophage, characterised by *MS4A6A*, *CD163* and *CD163L*, thus it was referred to as C0-Res. Several marker genes of cluster-1 were associated with tumorigenesis, such as *PARD3*[46], *EGFR*[47], and *SMAD3*[48], was thus labelled as C1-TAM (Table S4). The third subtype showed the significant upregulation of the signatures of M2-like MΦ, including *SLC16A10*[49], *SLC11A1*[50] and *CTSL*[51], and thus we named it C2-M2. The fourth subtype showed the marker genes of both dendritic cells (*ADAM19*, *HDAC9*, and *MCOLN2*)[52–54] and macrophages (*SLC8A1*, *RUNX3*, and *LCP1*)[55–57], which was referred to as C3-DC. The fifth subtype had several marker genes (*SEMA3A*, *ESRRG*, and *IL18*)[58–60] representing M1-like MΦ, which was labelled as C4-M1. The enrichment of biological processes (BPs) in each subtype was analysed (Fig. S5). More immune response associated BPs were observed in C2-M2, C3-DC and C4-M1, with several inflammation relevant processes only present in the C4-M1, such as ‘interleukin-21-mediated signalling pathway’, ‘inflammatory response’ and ‘regulation of cytokine production’. C1-TAM was highly enriched with cell development and tissue growth processes, and occupied a higher proportion in the CCII group, while other subtypes were more abundant in the CCI group (Fig. S6A).

The DEGs relevant to immune response appeared mostly upregulated in all the subtypes of the CCI group, especially in C0-Res, C1-TAM and C2-M2 (Fig. S6B). The average expression of selected genes associated with macrophage functions was compared across the subtypes, showing that *STAT1*, *HLA-DRB1*, *TMSB4X*, *C1QC* and *FGL2* were upregulated in all subtypes of the CCI group with a higher percentage of expression (Fig. 3B). Although *S100A8*, *DUSP1*, *MT2A*, and *JUNB* had higher average expression in the subtypes of the CCII group, their percentage of expression was comparatively low. The expression of the genes related to cell proliferation and extracellular matrix development, such as *CCN2*, *AEBP1*, *MGP*, and several members of the collagen family, was highly elevated in all subtypes of the CCII group. Notably, the upregulation of several genes encoding mitochondrial proteins associated with oxidative phosphorylation, for instance, *MT-CYO2*, *MT-CYO3*, *MT-ND4* and *MT-ND1*, were observed in the C0-Res, C1-TAM and C2-M2 of the CCII group (Table S4), which might be associated with active cellular metabolism during tumour growth. We then projected MΦs onto the two-dimensional state-space defined by Monocle3 for pseudotime analysis, to infer lineage trajectory for MΦ development (Fig. 3C). The trajectory began with the C0-Res and C2-M2, and then developed to separate directions, with one direction leading to C3-DC and C4-M1, while the other direction eventually linked to C1-TAM with a branch composed of C1-TAM, C3-DC and C4-M1. It appeared that the C3-DC and C4-M1 had similar developmental lineage along the pseudotime.

A total of nine states were thus derived from the trajectory, with large proportions of C0-Res and C2-M2 cells detected at State-1, State-3, and State-5, while C1-TAM cells gradually became the largest population at late pseudotime (Fig. S6C). There were high proportion of C3-DC in State-2, 4 and 9. It was evident C0-Res, C1-TAM and C2-M2 cells aligned more with the cells derived from the CCI group relative to the CCII group, except for State-9. The DEGs of each state were compared between the CCI and CCII groups (Table S4), and the macrophage marker genes showed higher percentage and/or average expression in most states of the CCI group, except for State-3 showing the upregulation of *CXCR4* and *DOCK8* in the CCII group (Figure S6D). The expression of mitochondria-associated marker genes and several collagens were elevated along the pseudotime, particularly for the State-5, 6, 7, 8 and 9 (Fig. S6E). The composition of each state within each subtype was displayed; C4-M1 and C2-M2 MΦs were respectively dominated by one cell state, whose population was mostly derived from the CCI group. State-4 and 9 cells occupied nearly 90% of C3-DC (Figure S6F).

There were four branches present along the pseudotime. Analysing the genes that were significantly dependent on Branch-1, we found upregulated expression of immune response relevant genes in State-1 and 2 cells, such as *CXCL9*, *CXCL10*, *IL15*, *IL18*, *DOCK8*, *DOCK10*, and *STAT1* (Fig. S7A). The expression of *DOCK8* and *STAT1* was significantly upregulated in State-1 cells of the CCI group, and so were *IFI44L*, *IFI44* and *IFNGR2*, which resulted in the activation of IFN-γ response. The genes largely downregulated in the other states post-branching were mainly expressed by M2-like MΦs, playing roles in various metabolisms, whereas resident-like MΦs appeared more active in cell proliferation. In terms of Brach-2, many DEGs in State-1, 3 and 4 were derived from the CCI group and associated with innate immune system post-branching (Fig. S7B). State-6 cells had comparatively lower level of antigen processing and presentation post branching, as suggested by the downregulation of MHC class I proteins, such as *HLA-A*, *B* and C*,* as well as *HLA-DQA1*, *HLA-DRB1* and *HLA-DRA* (Fig. S7C). Separated by Branch-4, the genes associated with cell adhesion and development were upregulated in State-8, which was more representative in the CCII group; State-8 cells of the CCI group exhibited functions in ion import and response to cytokine (Table S4). The genes relevant to chemokine production and neutrophil activation showed higher expression post-branching, such as *DENND1B*, *NLRP3*, *CD53*, *FGR*, *PTPRC*, *MNDA*, and *CD58* (Fig. S7D). This largely reflected the activation of cytokine-mediated signalling in the State-9 cells of the CCI group; in contrast, ECM-receptor interaction and PI3K-Akt signalling were more pronounced in CCII State-9 cells.

### The function of T cells was suppressed in the TME of CCII patients

**Fig. 4:**
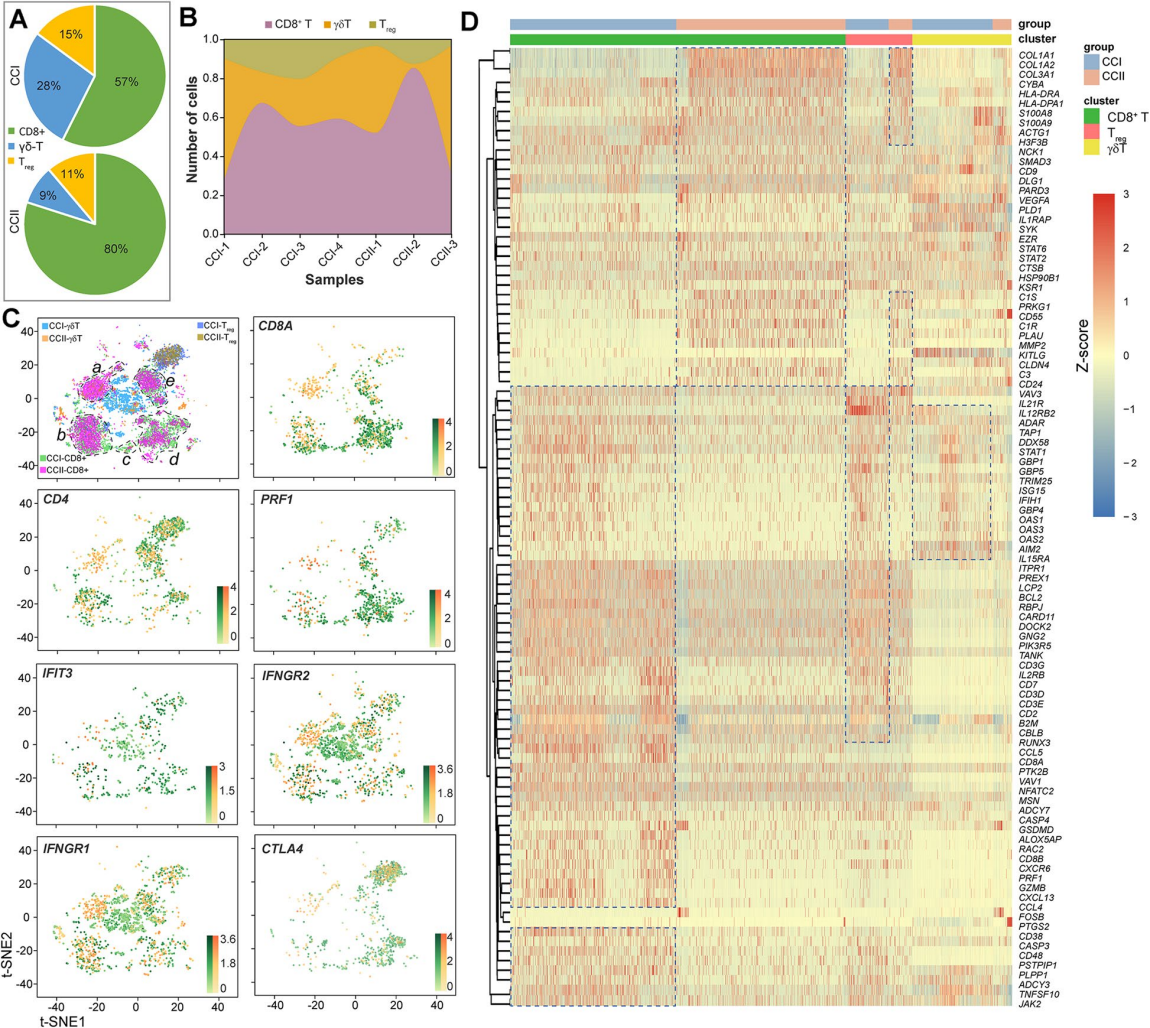
T cells were less activated in the CCII group patients. (**A**) The comparison of the proportions of CD8^+^, γδT and T_reg_ cells of entire T cell populations of the CCI and CCII groups. (**B**) The number of three T cell populations detected in the TME of individual patients. (**C**) The 2D-tSNE graphs comparatively display the distributions of different T cells, and the expression (in normalised value) of selected marker genes (incl. *CD8A*, *CD4*, *PRF1*, *IFIT3*, *IFNGR1*, *IFNGR1*, and *CTLA4*) in the CCI and CCII groups. (**D**) The hierarchical clustering of the top 100 DEGs between the three T cell types of the two groups (FC > 1.5 and *P*-value < 0.05)

Three populations of T cells were identified, including CD8^+^ T, γδT and T_reg_ cells. The proportion of CD8^+^ T cells was much higher in the CCII group (12.85%) than that in the CCI group (8.58%) (Table S1). Considering the entire T cell population, the populations of CD8^+^ T and γδT cells were largely modulated at different stages, with the former increased from 57% (CCI) to 80% (CCII) while the latter reduced by nearly 70% (from 28% in CCI to 9% in CCII) (Fig. 4A). The patients of the CCI group had a higher number of T_reg_ cells than the CCII group (Fig. 4B). The distribution of three T cell populations between the two groups was displayed in 2D-tSNE space (Fig. 4C). It was evident that CD8^+^ T cells separated into five major subtypes (a, b, c, d, and e), with subtype-*a* possessing a significantly higher number of cells derived from the CCII group. The CCI group had more cells derived from subtype-*b*, *c, d,* and *e*. The expression of selected maker genes characterising T_reg_ cells was compared between the CCI and CCII groups (Fig. 4C). *CD8A* and *PRF1* were highly expressed by the subcluster-*d* cells of CD8^+^ T, while the cells expressing elevated levels of *CD4* and *CTLA4* were mainly detected in T_reg_ cells of the CCI group. The expression of *IFNGR1* and *IFNGR2* was more expressed in γδT cells, whereas *IFIT3* was upregulated in the T_reg_ cells of the CCI group.

The hierarchical clustering of the top 100 DEGs across the three T cell populations of the two groups was displayed in Fig. 4D. The expression of DEGs associated with the activation of T cell immune response was upregulated in CD8^+^ T of the CCI group significantly, such as *CD7*, *RUNX3*, *CD3D*, *CD3G*, *GZMB*, *CD8B*, and *CCL5*. This was also partially observed for the T_reg_ cells. Several genes closely related to interferon response were upregulated in the CCI γδT cells, for example, *IFIH1*, *ISG15*, *GBP1*, *GBP4*, *GBP5*, *OAS1*, *OAS2* and *OAS3*. Notably, the expression of two genes (*CD38* and *CD48*) related to antigen-presenting were elevated in the CD8^+^ T and T_reg_ cells, but downregulated in the γδT cells of the CCI group. The genes regulating complement and coagulation cascades, and inflammation were clustered and showed higher expression in CD8^+^ T and T_reg_ cells of the CCII group, for instance, *S100A8*, *S100A9*, *CYBA*, *CD55*, *C3*, and *PLAU*. These observations meant that, compared to the CCI group, the T cells of the CCII group were more immunosuppressive, although the population of CD8^+^ T was significantly higher.

### B cells and DCs in the TME of CCII showed suppressed MHC class I antigen process

**Fig. 5:**
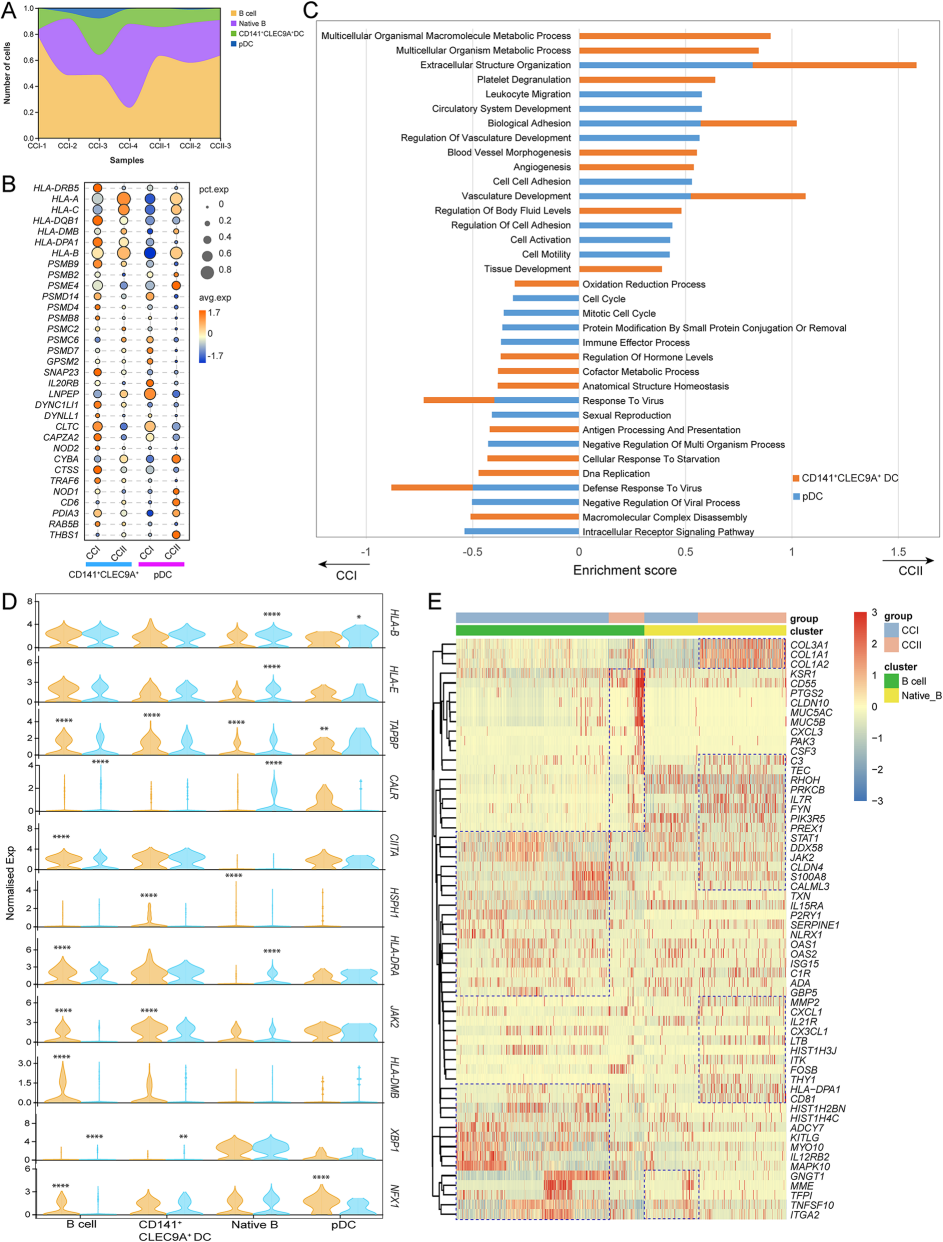
The B cells and DCs in the TME of the CCI group exhibiting enhanced MHC class-I pathway. (**A**) The proportions of B cells and DC cells detected in the CCI and CCII groups. (**B**) Comparison of the average expression of the genes associated with antigen presentation in the CD141^+^CLEC9A^+^ and pDC populations, with respect to their percentage of expression. (**C**) The top 25 biological processes enriched in CD141^+^CLEC9A^+^ and pDC populations. (**D**) Comparison of the normalised expression of selected genes associated with antigen presenting processes in B cells and DCs in the two groups. Student’s t-test was used to evaluate the significance (*: *P* < 0.05, **: *P* < 0.01, and ****: *P* < 0.0001). (**E**) The hierarchical clustering of the top 60 DEGs identified in the B cell populations between the two groups

The CCI group had a higher population of pDCs, while the population of B cells was more abundant in the CCII group (Fig. 5A). For both CD141^+^CLECL9A^+^ DCs and pDCs, the DEGs associated with antigen-presenting processes, such as *HLA-DRB5*, *HLA-DQB1*, and *HLA-DPA1*, were upregulated in the CCI group, whereas the CCII group had a higher average expression of *HLA-B*, *HLA-A*, *HLA-C*, and *HLA-DMB* (Fig. 5B). In addition, several members of the proteasome subunit gene family appeared more pronounced in terms of both average expression and percentage of expression in pDCs of the CCI group, for instance, *PSMB2*, *PSMD14, PMSC6*, and *PSM8*. The biological process analysis suggested that tissue development signalling was highly activated in the DCs of the CCII group, such as ‘angiogenesis’, ‘blood vessel morphogenesis’, ‘circulatory system development’, and ‘cell–cell adhesion’ (Fig. 5C). In contrast, many immune response processes were highly activated in the DCs of the CCI group, including ‘oxidation reduction process’, ‘immune effector process’, ‘response to virus’, ‘defence response to virus’, and ‘antigen processing and presentation’. It appeared the multicellular organism process was enriched in the CD141^+^CLECL9A^+^ DCs of the CCII group, but was suppressed significantly in the pDCs of the CCI group. The expression of selected DEGs associated with MHC class I antigen-processing was compared, and their upregulation in the B cells of the CCI group can be seen (Fig. 5D). The elevation of *COL3A1*, *COLA1* and *COLA2* was present in the native B cells of the CCII group (Fig. 5E). Two clusters of genes associated with the signalling of type I and II interferons were upregulated in B cells of the CCI group, such as *STAT1*, *OAS1*, *OAS2*, *ISG15*, *HLA-DPA1*, *IL12RB2*, and *JAK2*. The signalling of IL-18 and chemokine activity appeared more enhanced in the native B cells of the CCII group, supported by the higher expression of *CD81*, *MMP2*, *LTB*, *ITK*, and *CX3CL1*. The thymic stromal lymphopoietin (TSLP) and B cell receptor signalling were overrepresented by the upregulation of *TEC*, *FYN*, *IL7R*, and *STAT1* in the B cells of the CCII group.

### NK cells were more activated in the CCI group

**Fig. 6:**
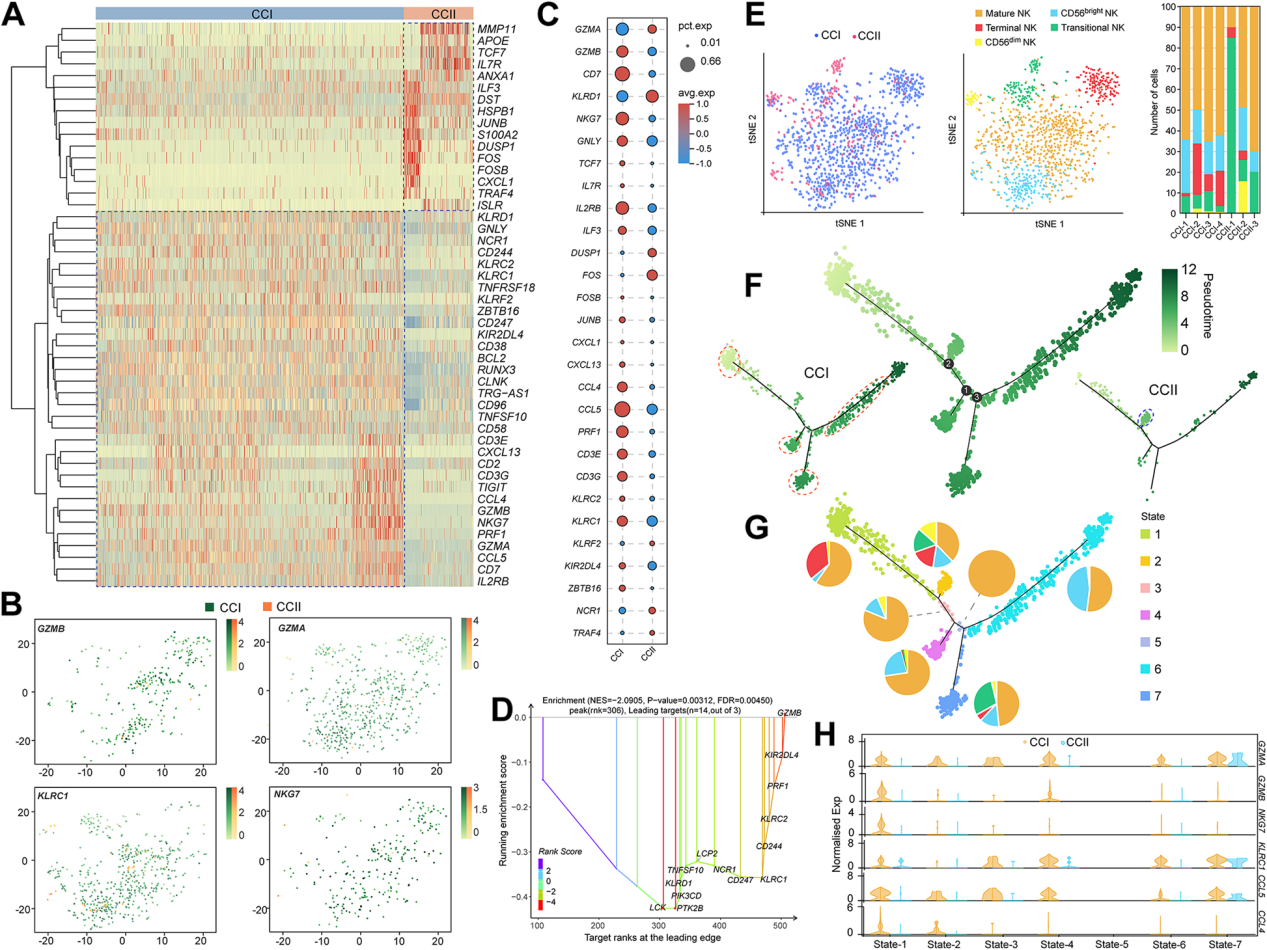
The function of NK cells was suppressed in the TME of CCII patients. (**A**) The hierarchical clustering of the top 50 DEGs of NK cells between the CCI and CCII groups. (**B**) The distribution of the NK cells expressing *GZMA*, *GZMB*, *NKG7*, and *KLRC1* in a 2D-tSNE space. (**C**) The comparison of the average expression of the genes associated with chemokine and cytokine signalling with respect to their percentage of expression in the two groups. (**D**) The ranking of the DEGs supporting the enrichment of natural killer cell mediated cytotoxicity pathway in the CCI group, with respect to the CCII group. (**E**) The 2D-tSNE graphs show the distribution of NK cells of the two groups and the subtypes, as well as the proportions of each subtype in individual patient. (**F**) Pseudotime trajectory analysis of NK cells. (**G**) The seven states identified along the pseudotime and their composition of the subtypes of NK cells. (**H**) Comparison of the normalised expression of the marker genes in each state of the two group, including *GZMA*, *GZMB*, *NKG7*, *KLRC1*, *CCL5* and *CCL4I*

The populations of NK cells occupied 2.52% and 0.85% of CCI and CCII cells, respectively (Table S1). The top 50 DEGs relevant to immune system and/or response KEGG pathways were compared, which clearly showed that most NK cells of the CCI group expressed higher levels of the marker genes positively associated with activated NK cell function, such as *KLRD1*, *KLRC1*, *GZMA*, *GZMB* and *NKG7* (Fig. 6A). The genes regulating IL17 and TNF signalling were highly expressed by the NK cells of the CCII group, including *TRAF4*, *FOSB*, *CXCL1*, and *FOS*; in addition, *HSPB1*, *APOE* and *IL7R*, which were related to the apoptosis due to altered *Notch3*, were more abundant and closely hierarchically clustered. The distribution of cells expressing selected marker genes of activated NK cells was displayed in a 2D-tSNE space, showing that *GZMA*, *KLRC1*, *GZMB* and *NKG7* were expressed only in a few NK cells of the CCII group (Fig. 6B). The DEGs associated with chemokine and cytokine signalling mostly had elevated average expression and percentage of expression in the CCI group, such as *CD7*, *IL2RB*, *CCL5*, and *CCL4* (Fig. 6C). The GSEA detected ‘natural killer cell mediated cytotoxicity’ as the only KEGG pathway enriched in the CCI group, supported by the upregulation of *GZMB*, *PRF1*, *KLRC1/2* and *CD244* (Fig. 6D). On the other hand, the enrichment of ‘ECM receptor interaction’ and ‘focal adhesion’ was detected in the CCII group (Table S5). In terms of Hallmark pathways, ‘INF-γ response’ and ‘allograft rejection’ were the only two pathways highly activated in the CCI group, whereas EMT and ‘coagulation’ were significantly enriched in the CCII group (Fig. S8A).

We then analysed the subtypes of the NK cells, which showed that the CCI group possessed higher populations of mature (characterised by the marker genes, *PRF1*, *GZMA*, *GZMB* and *CFL1*), CD56^bright^ (*KLRC1*, *NCAM1* and *GNLY*) and terminal (*WDR74*, *HIST1H1D*, and *HIST1H1E*) NK cells in general (Fig. 6E). The population of CD56^dim^ (*IL23R*, *IL7R*, and *TCF7*) NK cells was more present in the CCII group, so were the transitional (*FOS*, *FOSB*, and *JUNB*) NK cells. The development of NK cells in the two groups was inferred based on the trajectory analysis, where many cells present at early pseudotime belonged to in the CCI group, as well on the two branches along the time (Fig. 6F and Table S5). A total of seven states was thus identified, with higher proportion of mature NK cells detected in the CCI group (Fig. 6G). The proportion of terminal NK cells was the second largest in state-1, and then decreased in state-2 and 7 along the time, whereas the proportion of transitional NK cells increased. State-6 was mostly composed of CD56^bright^ and mature NK cells. The expression of the marker genes associated with the activated NK cells, including *GZMA*, *GZMB*, *NKG7*, *KLRC1*, *CCL5* and *CCL4*, was significantly upregulated in all states of the CCI group except for the state-5 (containing only mature NK), compared with the CCII group (Fig. 6H). The CCII group had more state-2 NK cells, while significantly less NK cells of the other states. For branch point 2, the CD56^bright^ cells of state-1 and 2 expressed higher level of DEGs associated with ‘natural killer cell mediated cytotoxicity’, such as *CD247*, *KLRC1*, and *NCAM1*, with respect to the other states post-branch (Fig. S8B). While the mature NK cells exhibited activated KEGG pathways related to tissue and cell development, such as ‘focal adhesion’, ‘regulation of actin cytoskeleton’, and ‘PI3K-Akt signalling pathway’, due to the upregulation of *ITGAT*, *ITGA1* and *BCL2* for state-1 and 2. Thus, in terms of immune response, CCI NK cells were more activated relative to those of the CCII group.

### Cancer cells of CCI and CCII patients showed distinct heterogeneity

**Fig. 7:**
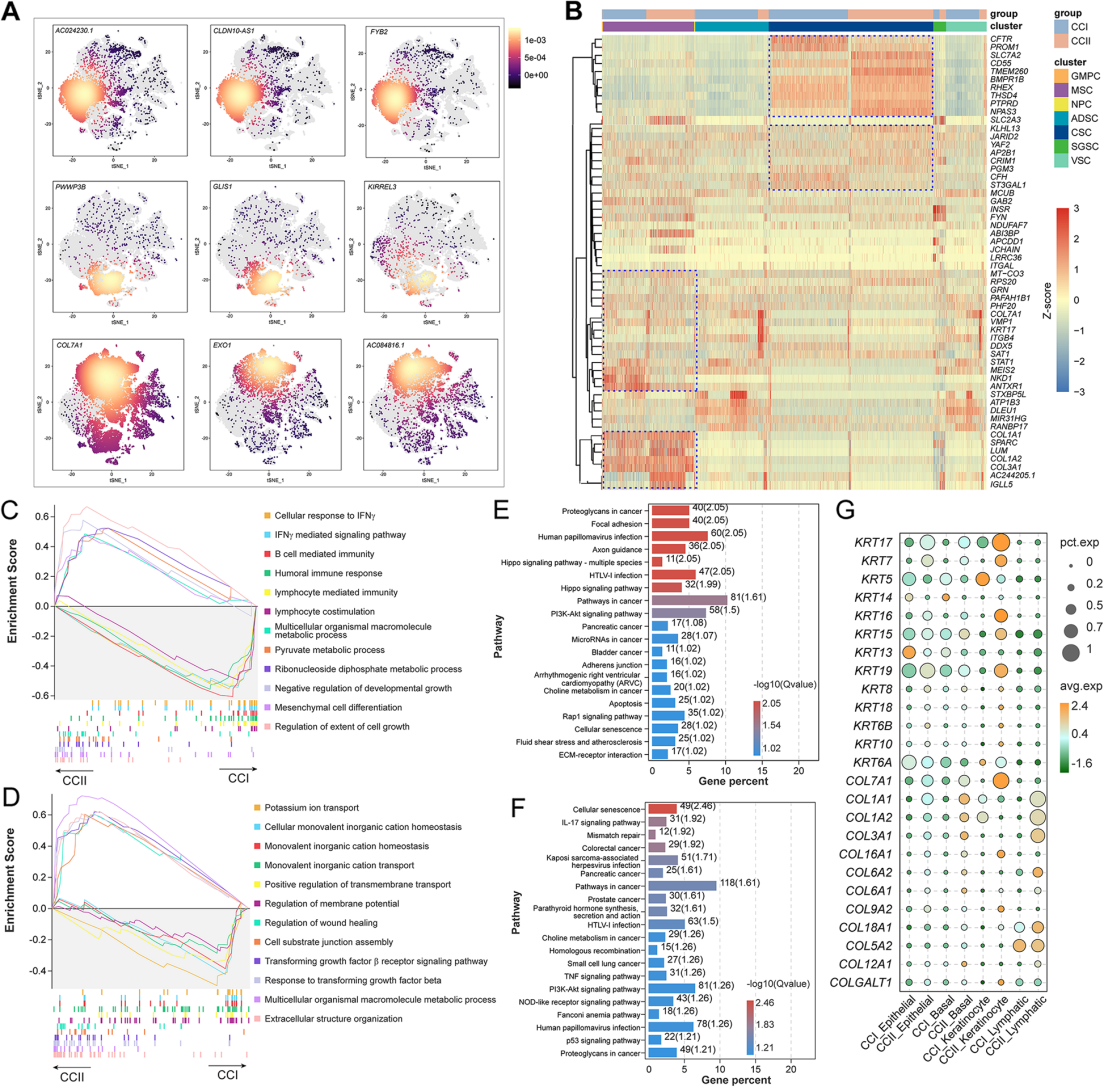
The tumour associated stem cells (TASCs) and epithelial cells were more immune response active in the TME of CCI patients. (**A**) The 2D-tSNE graphs show the expression density of selected marker genes of cancer stem cells (CSCs), mesenchymal stem cells (MSCs), adipose-derived stromal cells (ADSCs) and vascular stem cells (VSCs). (**B**) The heatmap hierarchically compares the top 10 DEGs of each stem cell-like population in the two groups. The GSEA of the top 6 biological processes enriched (*P*-value < 0.05) in the CSCs (**C**) and ADSCs (**D**) of CCI and CCII groups, respectively. The KEGG pathways enriched in the basal cells (**E**) and epithelial cells (**F**) of the CCI group. (**G**) The comparison of the average expression of keratin and collagen genes with respect to their percentage of expression in the two groups

Several other cell types associated with tumour progression, including stem cells and epithelial cells, were identified with the highest populations (Fig. 1; Table S1 and 2). The proportion of CSCs increased largely in the CCII group to 24.75%, compared to only 15.63% in the CCI group. Similarly, MSC population was higher (13.88%) in the CCII group. In contrast, the ADSCs showed lower proportion (3.13%) in the CCII group. The relative expression density of selected marker genes was displayed in a 2D-tSNE space (Fig. 7A). The top 3 marker genes of CSCs, including *CLDN10-AS1*, *AC024230.1* and *FYB2*, also showed certain expression in epithelial cells. A cluster of DEGs, such as *CFTR*, *PROM1*, *CD55*, and *RHEX*, were significantly upregulated in the CSCs and more pronounced in the CCII group, implying a high level of cell growth (Fig. 7B). The genes associated with inflammatory response, such as *STAT1*, *ITGB4*, *SAT1*, and *ANTXR1*, as well as several collagens, had higher expression in MSCs with respect to other stem cells. The GSEA identified that the top 6 pathways enriched in the CSCs of the CCI group were relevant to immune response, such as ‘cellular immune response to IFN-γ’, ‘IFN-γ mediated signalling’, and ‘B cell mediated immunity’ (Fig. 7C). In contrast, the pathways associated with cell growth were highly activated in the CCII CSCs. The signalling of TGFβ and extracellular matrix organisation appeared more enriched in ADSCs of the CCII group, whereas the activation of ion membrane transport was detected in those of the CCI group (Fig. 7D).

The signalling associated with multiple cancers was among the top KEGG pathways enriched in both basal and epithelial cells, with ‘human papillomavirus infection’ more present in the former (Fig. 7E and 7F). Since many genes expressing keratins and collagens were detected as the top DEGs of the four non-stem cell populations, the average expression of these genes with respect to their expression percentage were compared (Fig. 7G). Notably, the expression of collagens was upregulated in these cells of the CCII group in general, which was also observed for keratins except in the lymphatic endothelial cells. The keratinocytes showed high expression of most keratins and collagens. Furthermore, several members of collagen family were upregulated in multiple cell types of the CCII group, such as *COL1A1*, *COL1A2*, *COL3A1*, *COL5A1*, *COL5A2*, *COL6A1*, and *COL18A1* (Fig. S9A). The aberrant expression of many of them were positively correlated with the risk of cervical cancer, with the KM curves of four conveying significance, including *COL4A1*, *COL4A2*, *COL5A1*, and *COL12A1* (Fig. S9B), which implied their potential application in clinical diagnosis.

### Quantitative proteomic analysis revealed significant immunosuppression in CCII patients

**Fig. 8:**
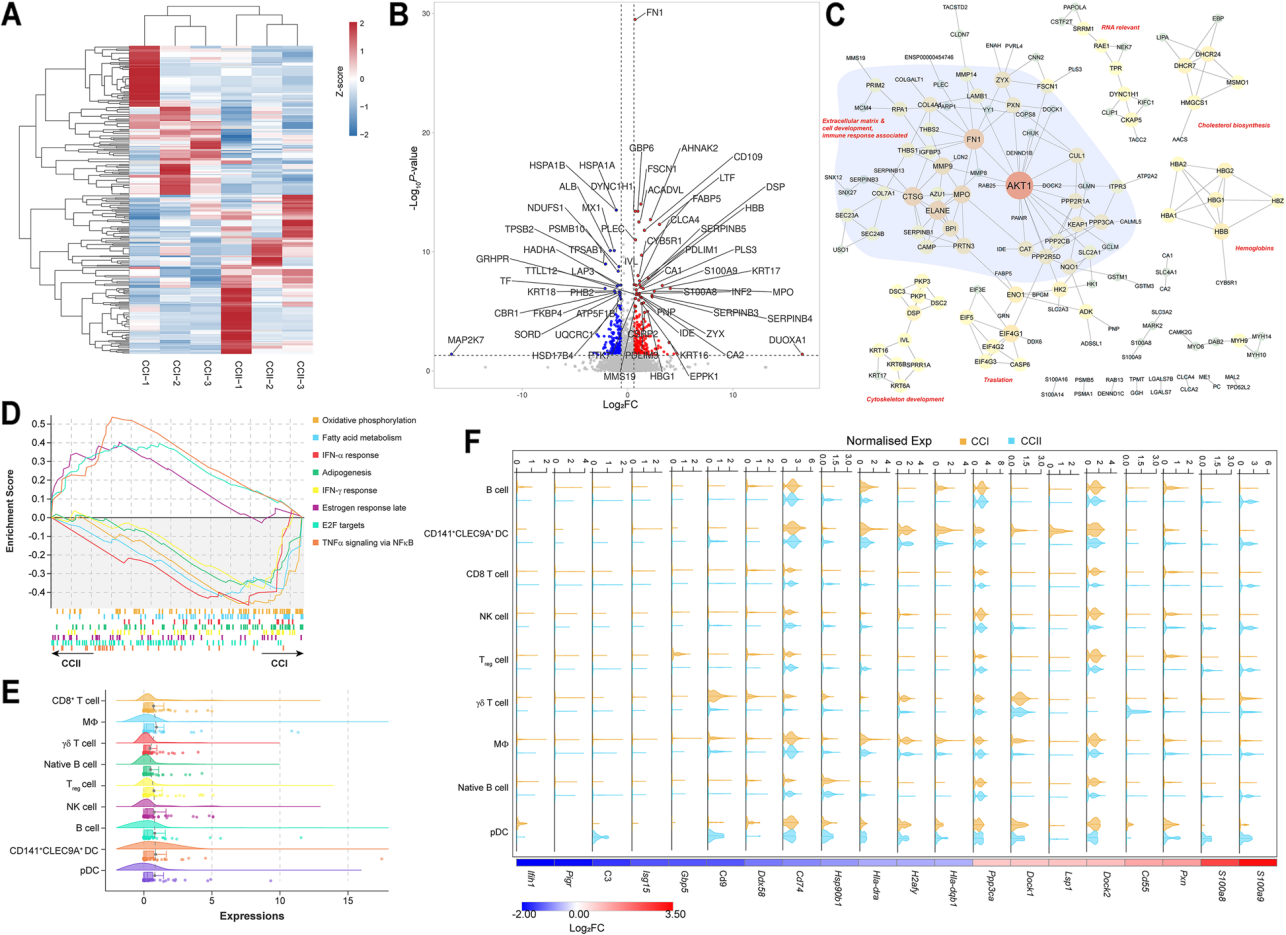
Quantitative proteomic comparison of the tumour tissues collected from the CCI (*n* = 3) and CCII (*n* = 3) patients. (**A**) Hierarchically clustering of DEP contents (in Z-score) identified from different patients. (**B**) Volcano graph displays the Log_2_FC versus -Log_10_*P*-value of the top 60 DEPs of the CCII relative to the CCI group. (**C**) The protein–protein interactions among the DEPs significantly upregulated in the CCII group. (**D**) The top 8 Hallmark pathways enriched in the two groups, predicted by the GSEA. (**E**) The expression profiles of the genes in different immune cells expressing the DEPs associated with INF-α response pathway identified in (**D**). (**F**) The correlation between the Log_2_FC values of the top 20 DEPs in the CCII group relevant to immune processes (based on GO terms) and their gene expression in different immune cell populations of the two groups

A total of 14,855 proteins belonging to 6,181 protein groups were identified from the CCI and CCII groups (Table S6). The PCA analysis showed a coverage of 55.0% and 29.6% of the difference between the CCI and CCI groups in PC1 and PC2, respectively (Fig. S10A), and the relatively close correlation between certain replicates of the two groups was present (Fig. S10B). The transcription factor (TF) ‘HMG’ was highly identified in the proteins corresponding to 73 genes, followed by ‘zf-C2H2’ (57) and ‘Homeobox’ (53) (Fig. S10C and Table S7). Notably, several TFs encoding proteins regulating cell development were identified, such as ‘MYB’, ‘CSD’ and ‘ARID’, as well as those relevant to immune response regulation, including ‘STAT’, ‘IRF’ and ‘NF-Y’. There were 541 differentially expressed proteins (DEPs) determined as quantifiable between two groups (Fig. 8A and Table S6), with the top 60 DEPs displayed comparatively with respect to the *P*-values (Fig. 8B). The DEPs significantly upregulated in the CCII group showed intensive interactions with high-confidence, with AKT1 (FC = 1.91) being the node with the highest degree, which interacts with several other high-degree nodes associated with the development of extracellular matrix, such as FN1, MMP9, PXN, CAT and CUL1 (Fig. 8C and Table S6). The interactions between MMP9 and the nodes related to immune response were present, for instance, CTSG, MPO, ELANE and AZU1. In addition, the downstream nodes interacting with CTSG included several members of SERPIN family and IGFBP3. The PPIs between DEPs upregulated in the CCI group showed a large cluster of histones (Fig. S10D). Several nodes regulating mitochondria-associated metabolism interacted with each other, such as CYC1, NDUFS1, NDUFS8 and UQCRC1. Importantly, three immune response relevant clusters were identified, which were composed of proteins related to antigen processing and presentation, stress response, and interferon response, respectively.

The GSEA revealed the enrichment of IFN-α (*P*-value = 2.2E-3) and IFN-β (*P*-value = 1.5E-2) response in the CCI group, whereas TNFα signalling via NFκB was enriched in the CCII group (Fig. 8D). Then, we compared the expression of genes encoding the DEPs detected on the IFN-α response pathway across the immune cells detected by snRNA-seq analysis, which showed that they were more expressed in MΦs, T_reg_, NK, B cells and DCs, with MΦs exhibiting the highest level of expression (Fig. 8E). The correlation between the protein profile and the cell types was assessed based on the FC values of DEPs and DEGs (Fig. S11). CSCs showed low correlation with all the other cell types and the proteome. The DEPs had the highest correlation (*P*-value < 0.001) with ADSCs, followed by NK cells and MΦs (*P*-value < 0.01). The FC values of the top 20 significantly regulated proteins in the CCII group relevant to immune processes (based on GO terms) were correlated to the expressions of their genes in different immune cell populations (Fig. 8F and Table S7). The expression of *S100A8* and *S100A9* was upregulated in most cell types of the CCII group, while *CD55* and *PXN* was remarkably upregulated in γδT cells. Thus, overall, a more immunosuppressive TME was formed in the CCII with respect to the CCI patients.

## Discussion

This study comparatively investigated the cell heterogeneity and functions within the TME of stage-I and II CC patients using snRNA-seq analysis. We found that the CCI patients had MΦs showing proinflammatory function, as indicated by significantly activated IFN-α and IFN-γ response signalling, whereas the MΦs of the CCII patients exhibited the activation of many pathways related to cell growth and tissue development. The CD8^+^ T cells appeared more activated in the CCI group with a lower population, and the populations of T_reg_ and γδT were largely reduced in the CCII group. Immune response, particularly the MHC class-I pathway, was more pronounced in the DCs and B cells of the CCI group, whereas metabolic and developmental processes were enriched in the CCII group. The proportion of NK cells was reduced more than 60% in the CCII relative to the CCI group, which had the upregulation of many DEGs marking the activation of NK cell function, especially for the mature and CD56^bright^ NK cells. The immune response relevant pathways were enriched in several stem cell types of the CCI group in general, while the cell and tissue growth, as well as metabolic pathways, were highly present in those of the CCII group. In addition, the quantitative proteomics revealed the activation of IFN-α and IFN-γ response signalling in the CCI group, which was accorded with the observation of snRNA-seq analysis.

Significant upregulations of more than 30 collagens were present in different cell types with snRNA-seq analysis. Collagens are major components of the TME and play roles in cancer fibrosis, as well as interacting with receptors, exosomes, and microRNAs to influence tumour cell behaviours [61]. It has been previously found that *COL1A1* was significantly elevated at both mRNA and protein level in CC tissues relative to normal tissues, and correlated negatively to radiosensitivity [62]. *COL6A1* was suggested as an oncogene in the initiation and progression of CC and a predictor for poor prognosis [63]. The high expression of *COL14A1* in residual CC after a 50-Gy dose of irradiation was detected by quantitative PCR [64]. *COL7A1* and *COL8A1* were identified bioinformatically as two of five genes associated with the collagen assembly that might be used as a single combinatorial prognostic marker for stage-II CC [65]. The role of *COL10A1* in CC progression was suggested [66]. The co-expression of *COL1A1*, *COL4A1*, *COL5A1*, *COL5A2* and *COL7A1* with a potential therapeutic target for CC *P4HA2*, was identified [67]. However, the correlation between the other collagens and CC, and their roles in progression remains largely unclear. The novel transcript *AC244205.1* was found as one of the most upregulated DEGs in many cell types of the CCII group, such as DCs, MΦs, T cells, MSCs, NPCs, and GMPCs, implying it may play a role in the progression of CC. A recent study has revealed that the upregulation of *AC244205.1* was observed in cholangiocarcinoma patients with better overall survival [68]. However, there is no report about the molecular function of *AC244205.1* in CC, which will be investigated in our future studies.

The expression of DEGs related to mitochondrial respiratory machinery, such as *MT-CYB*, *MT-CO2*, *MT-CO3*, *MT-ND1*, *MT-ND2*, and *MT-ND4*, were elevated in multiple cell types of the CCII group. This implied the activation of cytochromes on the respiratory chain, resulting in high level of mitochondrial biogenesis, which might be due to the significant energy consumption for tumour cell growth at stage-II with respect to stage-I, such as EMT, ‘myogenesis’, ‘hypoxia’, as well as multiple metabolic processes, suggested by the snRNA-seq analysis. A previous study showed that the inhibition of mitochondrial complex III, subsequently affecting mitochondrial respiration by atovaquone, can inhibit the proliferation and induce apoptosis in certain CC cell lines in vitro and in vivo on a mouse model [69]. The impairment of mitochondrial function via interfering certain signalling pathways, such as mTOR and CaMKII/Parkin/mitophagy, was targeted by several studies to tackle metabolic stress in CC cell lines, to inhibit the cancer cell growth [70–72]. Our study provided more target genes for interfering upregulated mitochondria activity in CC.

Compared with the CCI group, the snRNA-seq analysis found immunosuppression in nearly all immune cells of the CCII group, which led to an overall immunosuppressive TME collectively. *IGLL5* was the DEG upregulated in the MΦs of the CCII group following the collagens and mitochondrial genes. It has been recently shown to be closely correlated with tumour‐infiltrating immune cells, including MΦs, in clear cell renal cell carcinoma based the TCGA data [73]. The fusion of *IGLL5* was suggested to promote metastasis of the lymph nodes and play a role in breast cancer development [74], though its role in MΦs in the TME of CC remains unclear. *MMP11* was identified nearly unique to the MΦs of the CCII group. The immunotherapeutic role of *MMP11* in different cancers has been suggested previously [75–77]. Its overexpression was characterised in CC cell lines [78] and in cervical precursor lesions [79]. In breast cancer, *MMP11* expression was considered as a biomarker for prognosis [80], and correlated with a high CD68/(CD3 + CD20) ratio in CD68^+^ macrophages [81], which caused the polarisation of macrophages in the tumour centre, resulting in a higher metastatic phenotype [82]. This implies a similar process involved *MMP11* might occur in the TME of the CCII group.

Besides, *CCL19*, *IL7R*, *SPARC*, *MGP*, *LUM*, and *CXCR4* were highly expressed in the MΦs of the CCII group, and in each MΦ subtype. The overexpression of *CCL19* was found in CC cell lines and patient tissues, and its knockdown via siRNA inhibited the proliferation of CC cells in vitro via apoptosis pathway [83]. *SPARC* was associated with epithelial-mesenchymal transition and overexpressed in CC patients with poor prognosis [84], and was highly elevated in the CCII group. Increased MGP was significantly observed in high-grade cervical premalignant lesions with elevated hTERT mRNA expression [85]. In uterine CC tissues, *LUM* was expressed in most cancer cells and stromal fibroblasts, indicating its roles in the growth or invasion of CC [86]. The CXCL12/CXCR4 chemokine pathway was targeted for improving the therapeutic ratio in patient-derived CC models with radio-chemotherapeutic treatment [87].

Although there were higher number of CD8^+^ T cells in the CCII group, their function was largely suppressed compared with the CCI group. Many interferon-induced genes and receptors were expressed by γδT cells of the CCI group, while the population of γδT cells was very small in the CCII group. γδT cells link the innate with adaptive immunity, to protect the epithelium from trauma and infection, which have been suggested as potential therapeutics against HPV in patients [88]. The number of γδT cells was negatively associated with the progression of CC [89]. γδT cells alone were found to inhibit tumour growth, and if combined with galectin-1 antibody, they could provide a more effective immunotherapy for CC [90]. However, highly expressed HPV16 oncoproteins at the cancer stage induced a reorganisation of the local epithelial-associated γδT cell subpopulations, to promote angiogenesis and cancer development [91]. Thus, the significant reduction in the number of γδT cells may be associated with the high degree of tumorigenesis in stage-II CC.

The population of NK cells was remarkably reduced in the CCII group, so was the immune response, compared with those of the CCI group, which showed remarkably high activation of ‘INF-γ response’ and ‘allograft rejection’ pathways. The most enriched pathways in the CCII group were associated with cell organisation and tissue development, possibly correlated to more active tumour growth. This was particularly significant for the state-2 NK cells of the NK development, which showed the remarkable activation of EMT pathway (*P*-value *≈* 0). This was largely attributed to the upregulation of *FBN1*, *FN1*, *SERPINE1*, *VCAN*, and many collagens in the CCII group. Previous study discovered that collagens promote the accumulation of NK cells in Foci of infection near the lymph node capsule [92]. We thus speculate that the high abundance of collagens in NK cells might be cellular mechanism responding to extremely low NK cell number in the TME of stage-II CC, seeking to recruit more active NK cells.

The proteomic analysis revealed distinct protein profiles in the CCI and CCII groups. The activation of IFN-α and γ response in the CCI group was accorded with the comparative observation in multiple cells, especially immune cell types, by snRNA-seq analysis. The DEPs upregulated in the CCII group were largely associated with extracellular matrix, cell and tissue development, and metabolism, suggesting the higher degree of tumorigenesis. AKT1 was the node DEP with the highest degree of interactions. The signalling of PI3K/AKT/mTOR was found to regulate the virus/host cell crosstalk in HPV-positive CC [93], and the elevated level of pAKT is associated with radiation resistance in CC [94]. The inhibition of AKT, subsequently disrupting the signalling with mTOR, induced higher degree of cell death and decreased glucose uptake in CC [95]. FN1 was the most upregulated DEP interacting with multiple other DEPs of the CCII group, it was shown to promote the tumorigenesis of CC via activating FAK signalling pathway [96]. FN1 interacts with MPO, which has controversial role in different diseases [97], and a type of its gene polymorphism leads to reduced anti-tumour activity that may play a role in development of CC [98]. In terms of DEPs upregulation in the CCI group, there was a big cluster of histones intensively interacting with each other, including H2A/H2B and H4 members, which play important roles in transcription, DNA replication and repair [99]. It was identified that histone genes can be used as independent prognostic factors for survival prediction among CC patients, including HIST1H4A, HIST1H4E and HIST1H4K that were detected DEPs in this study. This implies that other histones might also be considered as markers, in combination with other DEPs upregulated in the CCI group, such as HSPs and interferon response relevant proteins, for the early detection of CC.

## Conclusions

In this study, we have comparatively investigated the TME of stage-I and II CC patients using snRNA-seq and label-free quantitative proteomic analysis. It was evident that the heterogeneity of nearly all immune cells of stage-II CC patients, including MΦs, T cells, B cells, NK cells and dendritic cells, were largely modulated, with their phenotypes to be significantly immunosuppressive. In contrast, these cells showed elevated immune response in stage-I CC patients, with INF-α and γ response being the most activated signalling pathways. Several histones showed the potential for diagnosing CC at an early stage. Notably, for the stage-II CC patients, the significant upregulation of collagens identified respectively by snRNA-seq analysis in multiple cell types and proteomic analysis, suggested that they might be used as the prognostic markers. For the first time, the correlation between aberrant expression of novel transcript *AC244205.1* and stage-II CC was revealed. These discoveries provide important clues for clinical diagnosis and immunotherapy of CC, which warrant further research.

## Supplementary Information

**Additional file 1.** Supplementary figures and supplementary table captions

**Additional file 2.** Supplementary tables

## Data Availability

The snRNA-seq data is available on the Institute Single Cell Portal [https://singlecell.broadinstitute.org/single_cell] under accession number SCP1950. The mass spectrometry proteomics data have been deposited to the ProteomeXchange Consortium via the PRIDE[100] partner repository with the dataset identifier PXD029103.

## Abbreviations

MΦ: Macrophage
TME: Tumour microenvironment
TCGA: The Cancer Genome Atlas
HPV: Human papillomavirus
scRNA-seq: Single-cell RNA sequencing
snRNA-seq: Single-nucleus RNA sequencing
DEG: Differentially expressed gene
DEP: Differentially expressed protein
TNF-α: Tumour necrosis factor-α
TAM: Tumour-associated macrophage
FIGO: The International Federation of Gynecology and Obstetrics
MHC: Major histocompatibility complex
IFN-α: Interferon alpha
IFN-β: Interferon alpha
IFN-γ: Interferon gamma
CCI: Cervical cancer stage-I
CCII: Cervical cancer stage-II
GSEA: Gene set enrichment analysis
TF: Transcription factor
