# Supplementary material for: Single-nucleus RNA sequencing and deep tissue proteomics reveal distinct tumour microenvironment in stage-I and II cervical cancer
Source: J Exp Clin Cancer Res. 2023 Jan 23;42:28. doi: 10.1186/s13046-023-02598-0 (PMC9869594; doi:10.1186/s13046-023-02598-0)
Supplement: Supplementary file 1 — Additional file 1. Supplementary figures and supplementary table captions [file 13046_2023_2598_MOESM1_ESM.pdf]

Supplementary Information

Supplementary figures

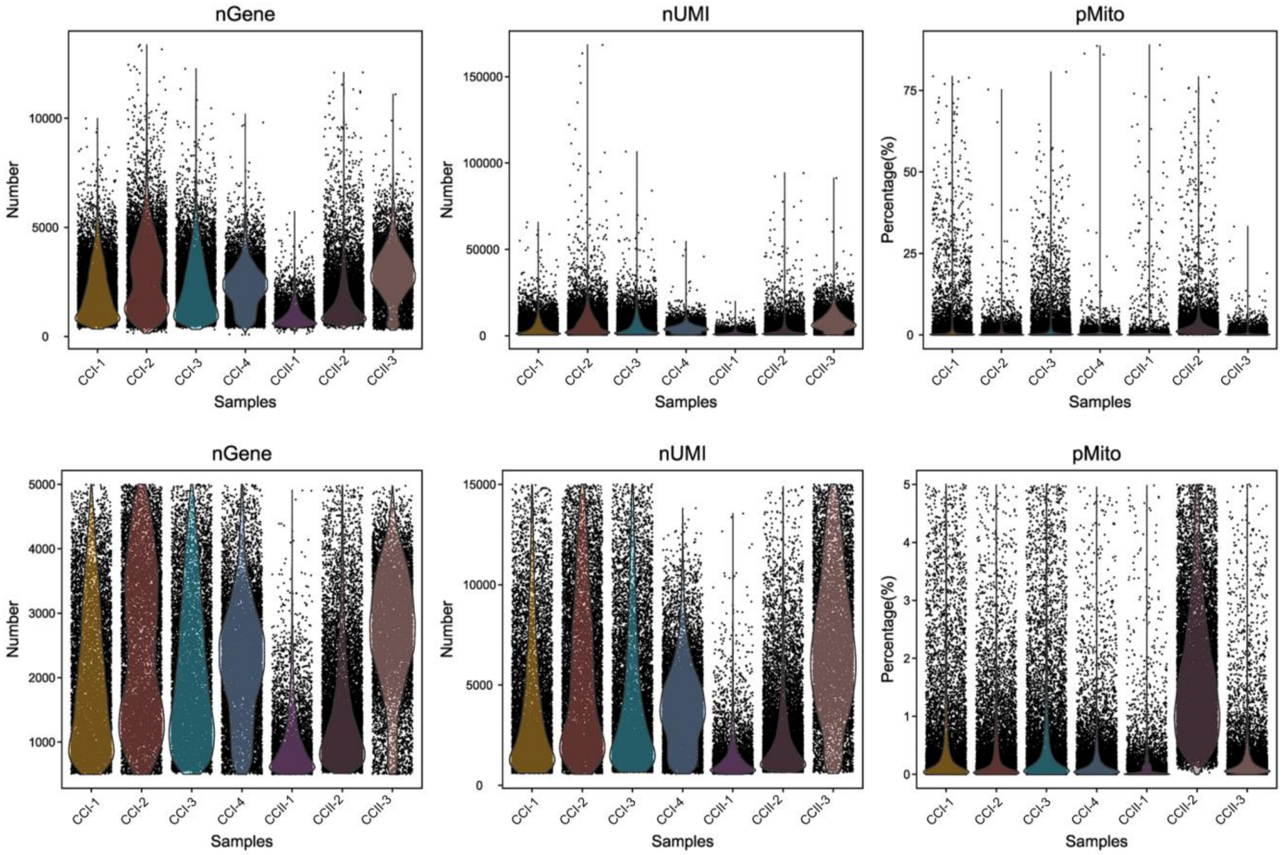

Fig. S1 Quality metrics including the number of unique molecular identifiers (UMI), genes detected per cell, and reads aligned to the human genome.

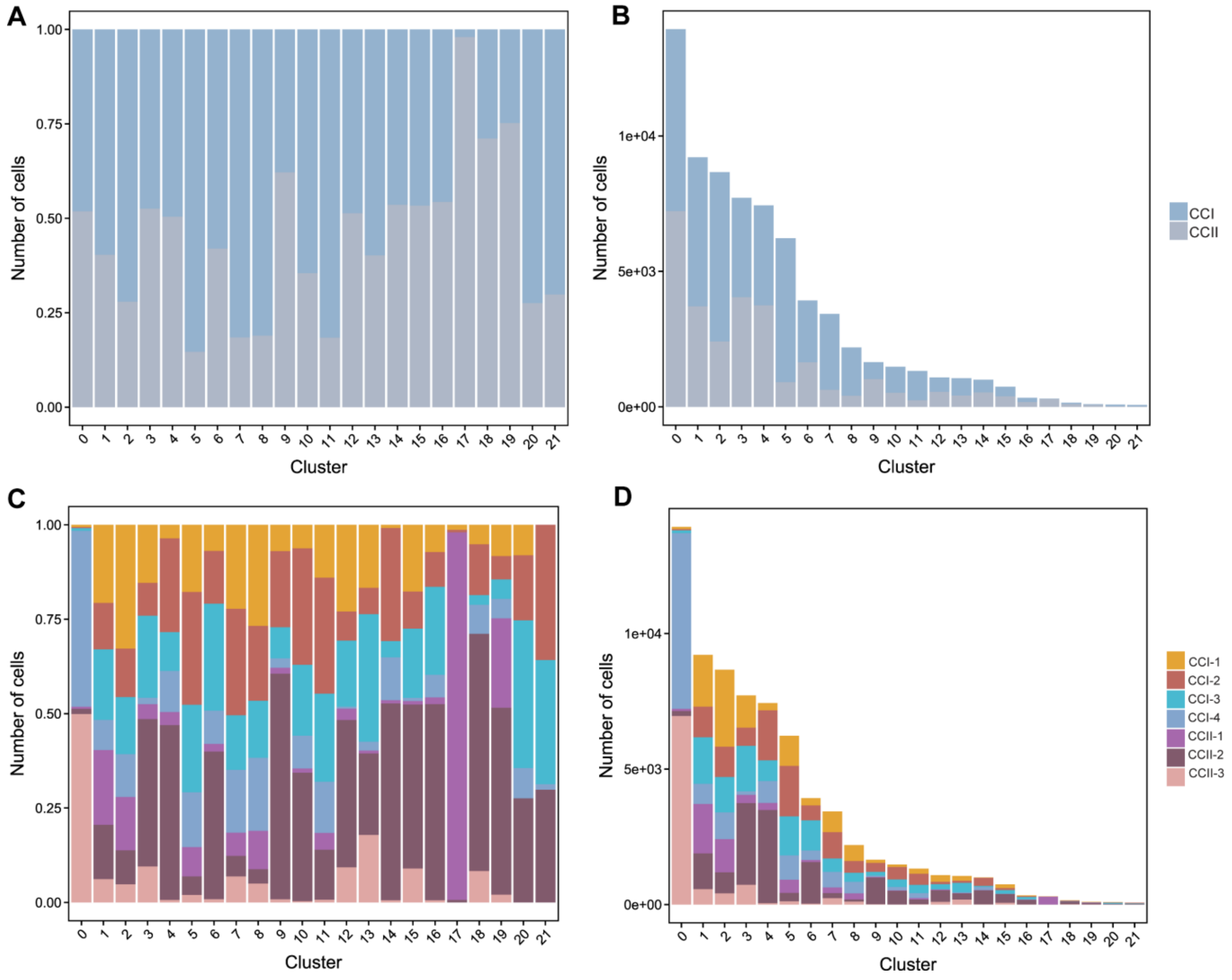

Fig. S2 The populations of different cell types detected in the CCI and CCII groups in percentage (**A**) and the number of cells (**B**). The individual patient's contribution to each cell type in percentage (**C**) and the number of cells (**D**).

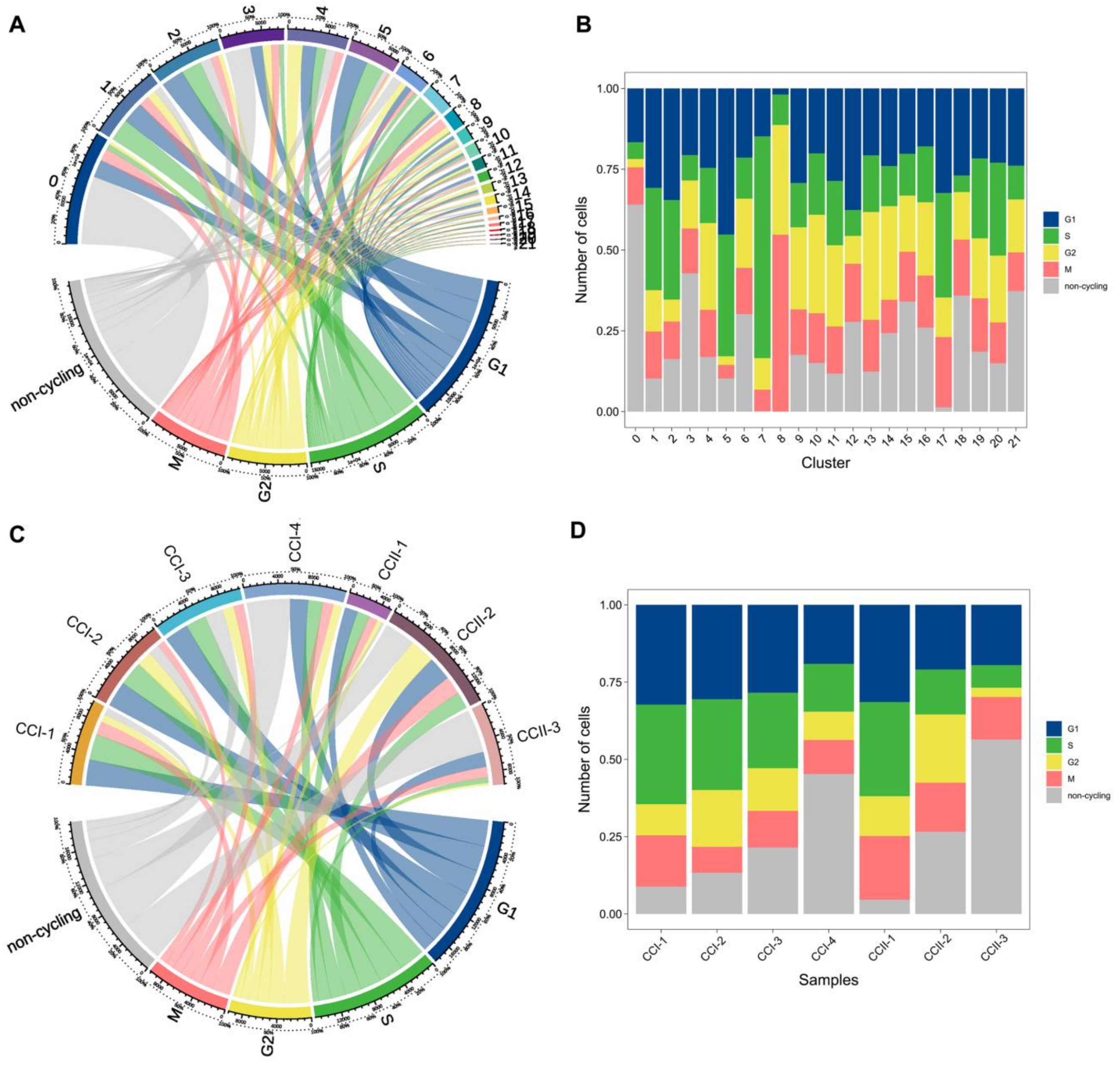

Fig. S3 Cell cycle analysis of different cell types and individual patients. (**A**) The correlation between 21 cell types and cell cycles; (**B**) The number of cells (in percentage) with different cell cycles in each cell type; (**C**) The correlation between the patients and cell cycles; (**D**) The number of cells (in percentage) with different cell cycles in each patient.

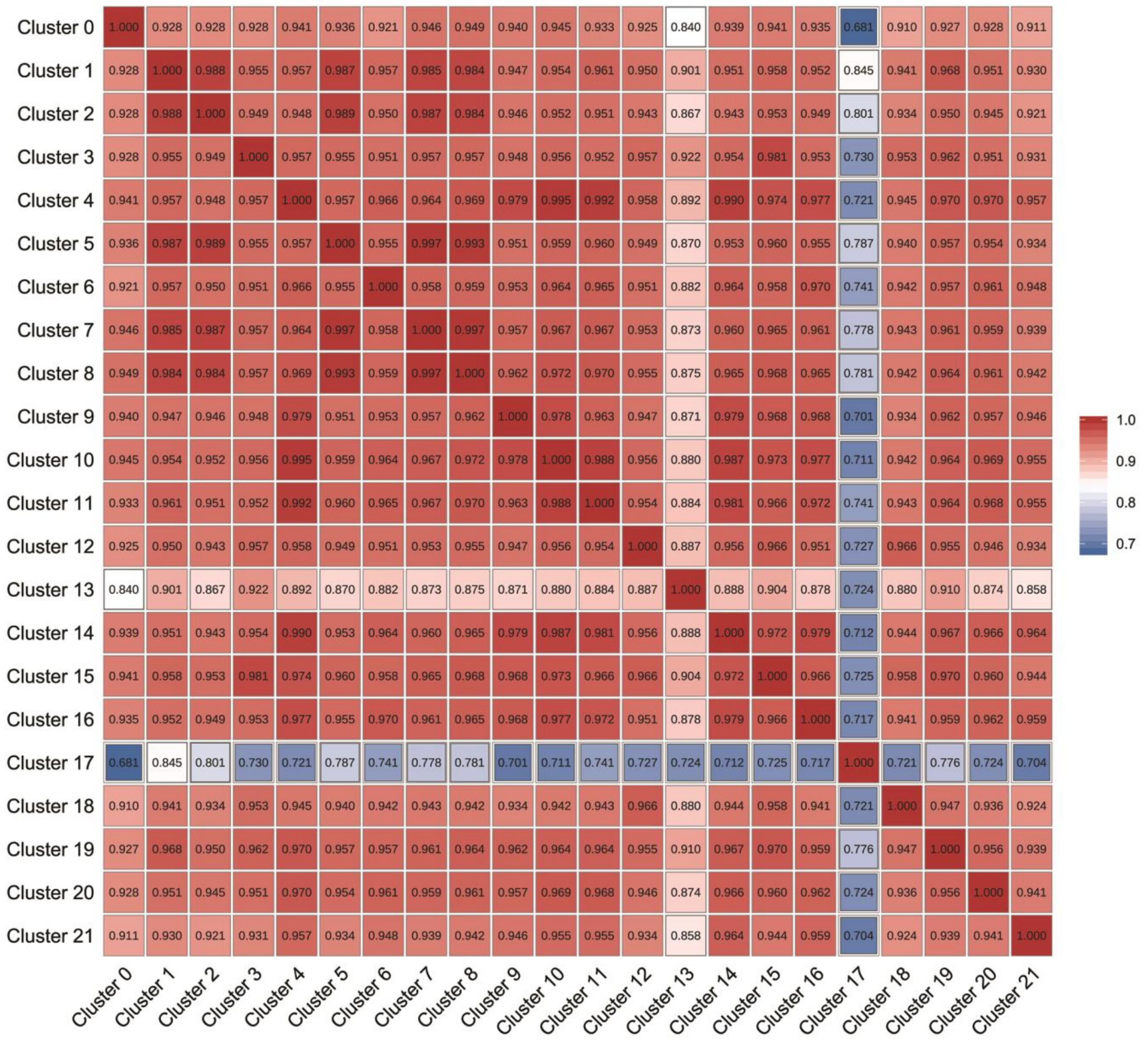

Fig. S4 The correlation between different cell types predicted based on gene expression.

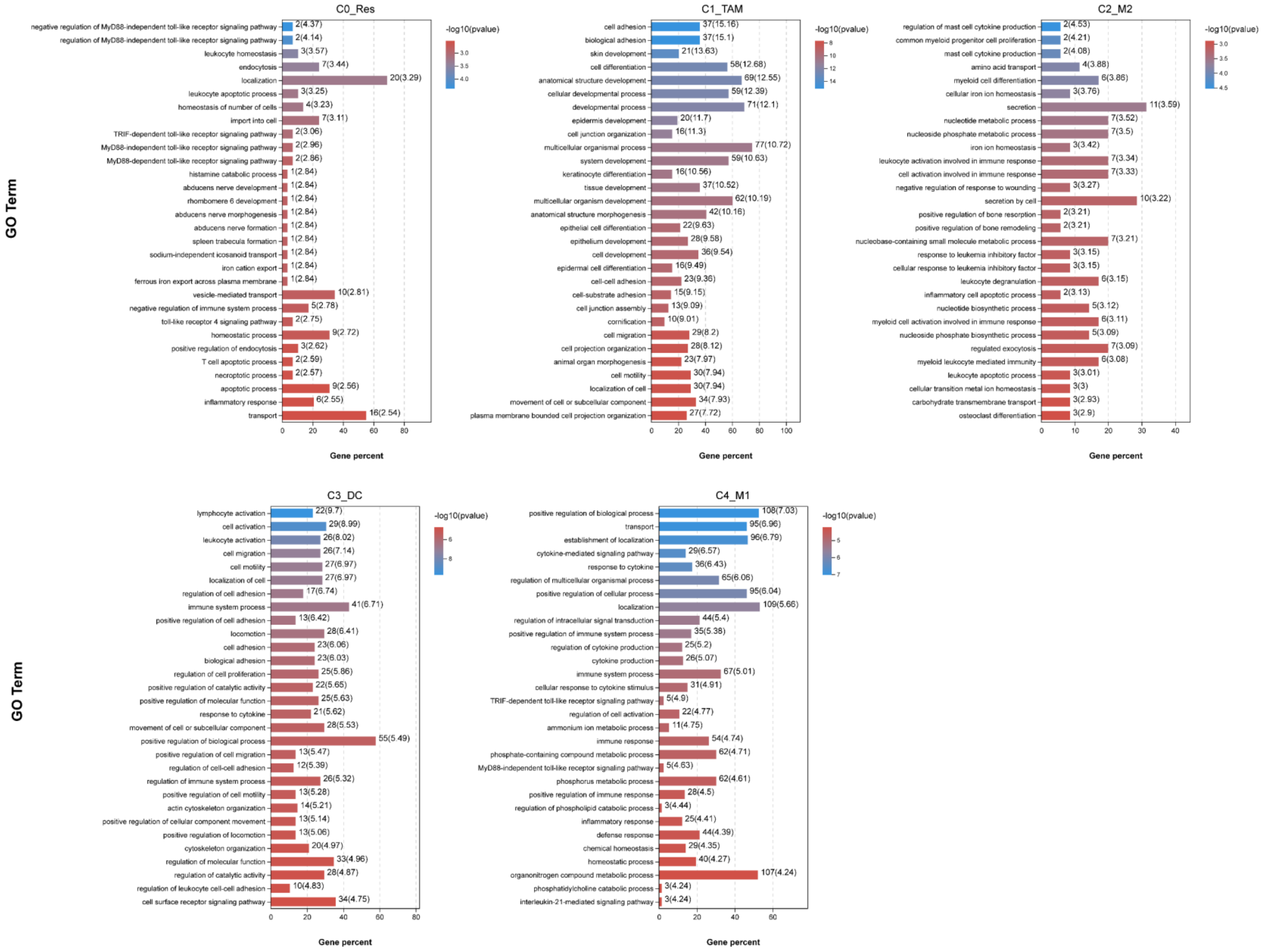

Fig. S5 Enrichment analysis of biological processes in subtypes of macrophages.

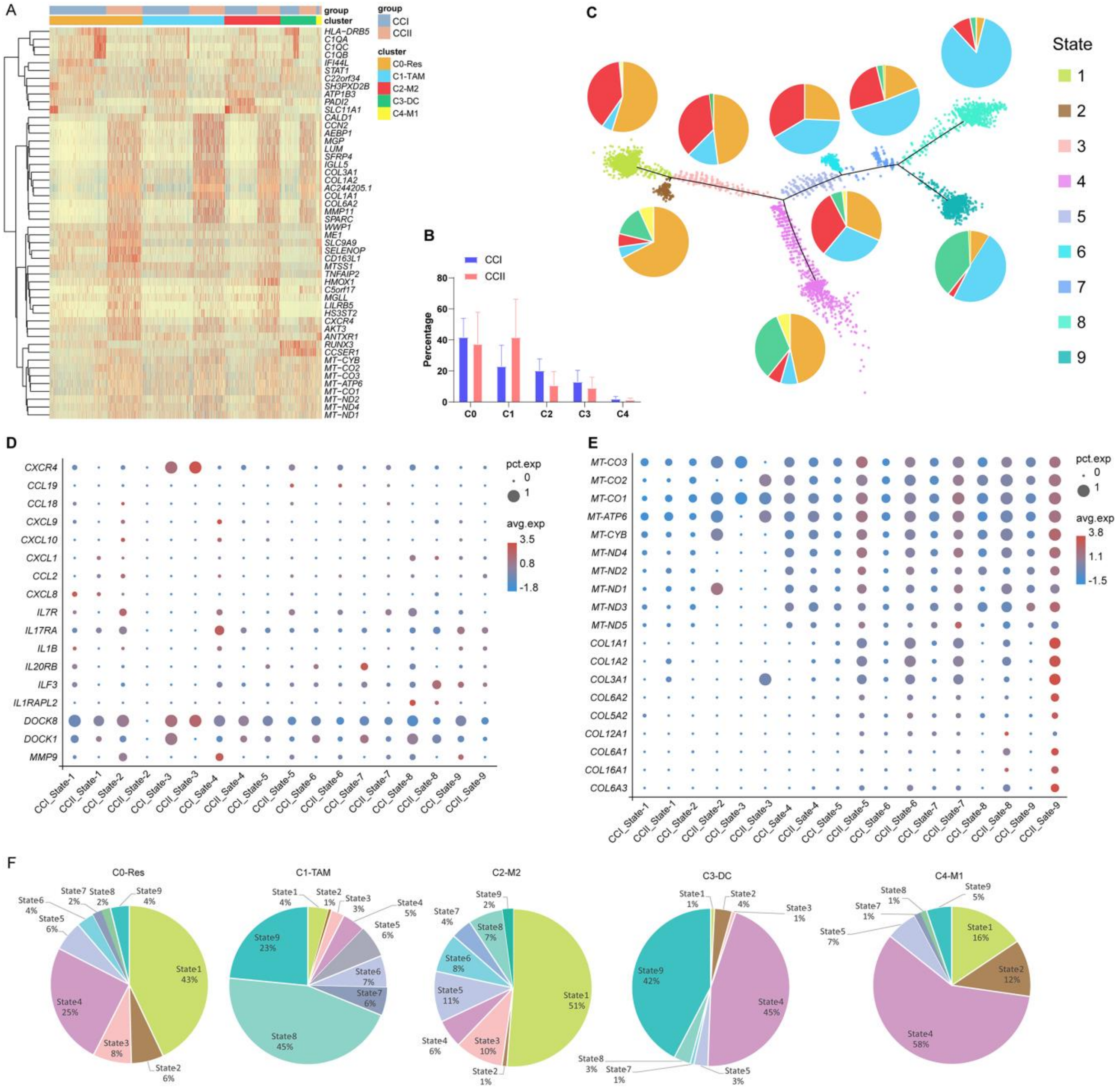

Fig. S6 Comparison of the macrophage subtype genotypes of CCI and CCII patients and the development analysis. (**A**) Hierarchical clustering of top 50 DEGs of subtypes; (**B**) The populations of MΦ subtypes of CCI and CCII groups; (**C**) Trajectory analysis of MΦ development shows nine states and four branching points; (**D**) The average expression of cytokine, chemokine, and interleukin genes of cells in different states of the CCI and CCII groups; (**E**) The average expression of mitochondria associated genes and collagens in different state cells of the CCI and CCII groups; (**F**) Composition of each state within each MΦ subtype.

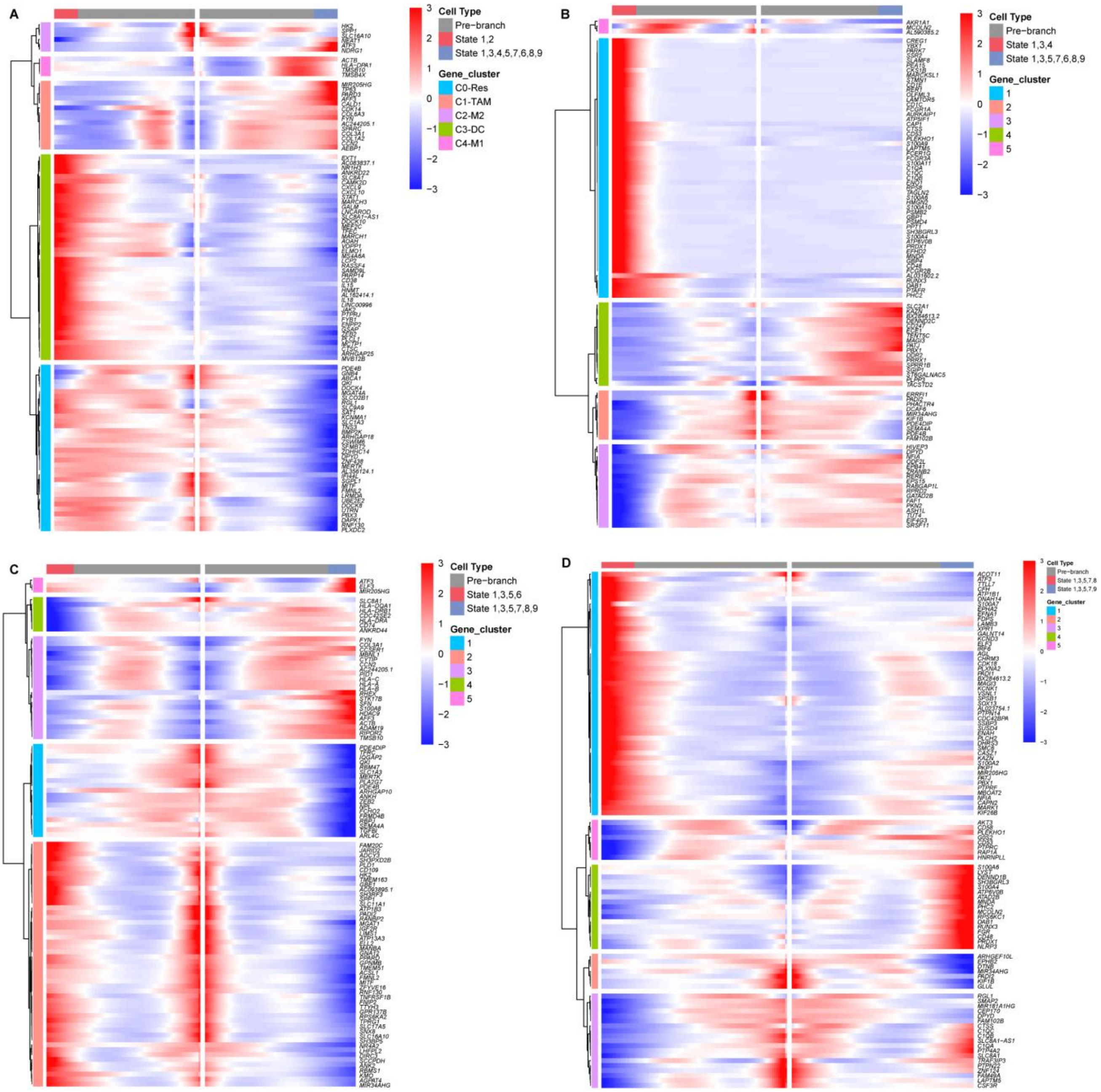

Fig. S7 Hierarchy clustering of the gene set with similar expression trend in different branches of the trajectory of MΦ cells. The horizontal axis is the pseudo-time point (the pseudo-time point gradually increases from left to right), the vertical axis is the gene expression level, and different colours indicate the level of gene expression level.

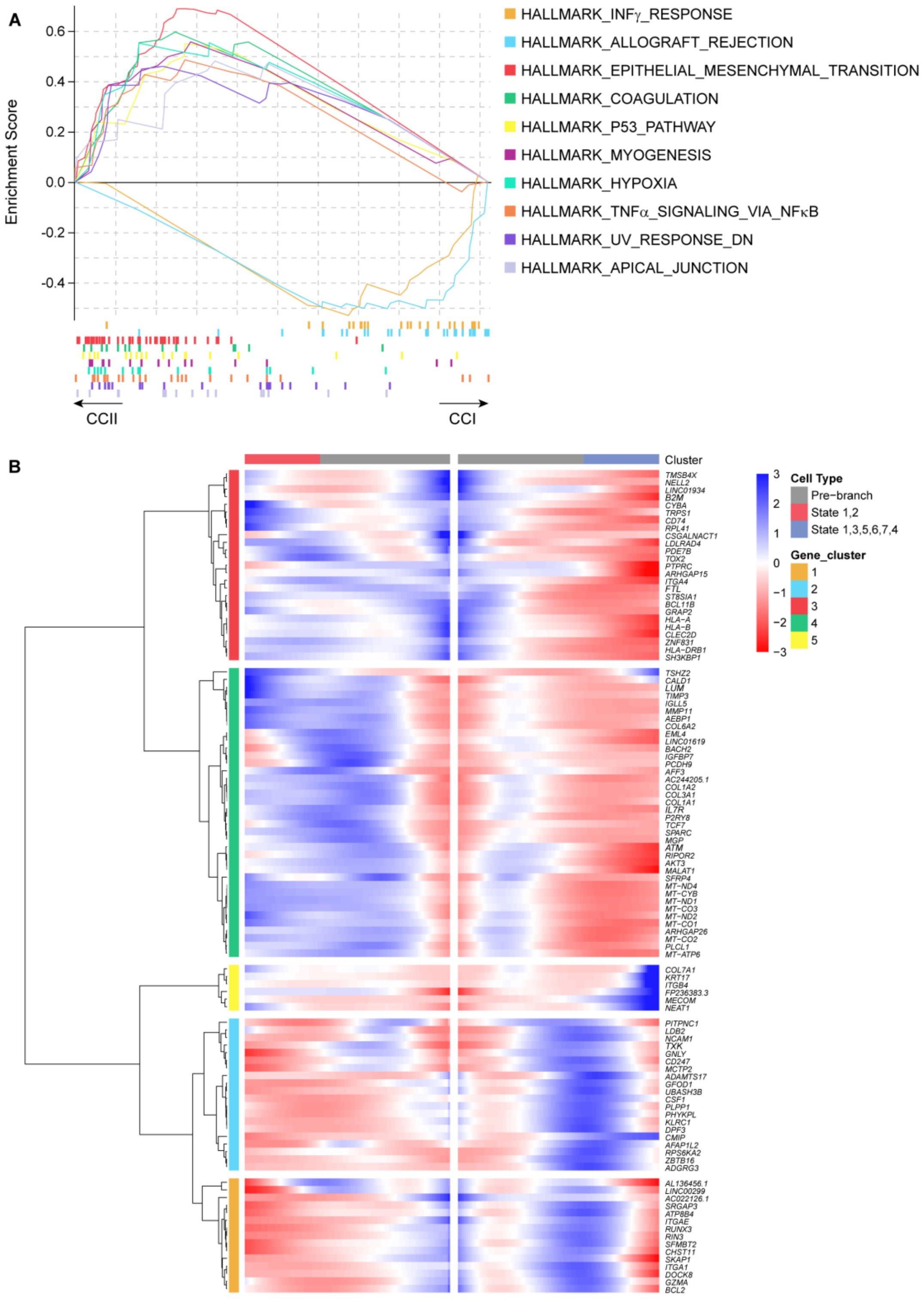

Fig. S8 (A) GSEA analysis of the Hallmark pathways enriched in the NK cells of the CCI and CCII groups. **(B)** Hierarchy clustering of the gene set with similar expression trend in branch point 2 of the trajectory of NK cells. The horizontal axis is the pseudo-time point (the pseudo-time point gradually increases from left to right), the vertical axis is the gene expression level, and different colours indicate the level of gene expression level.

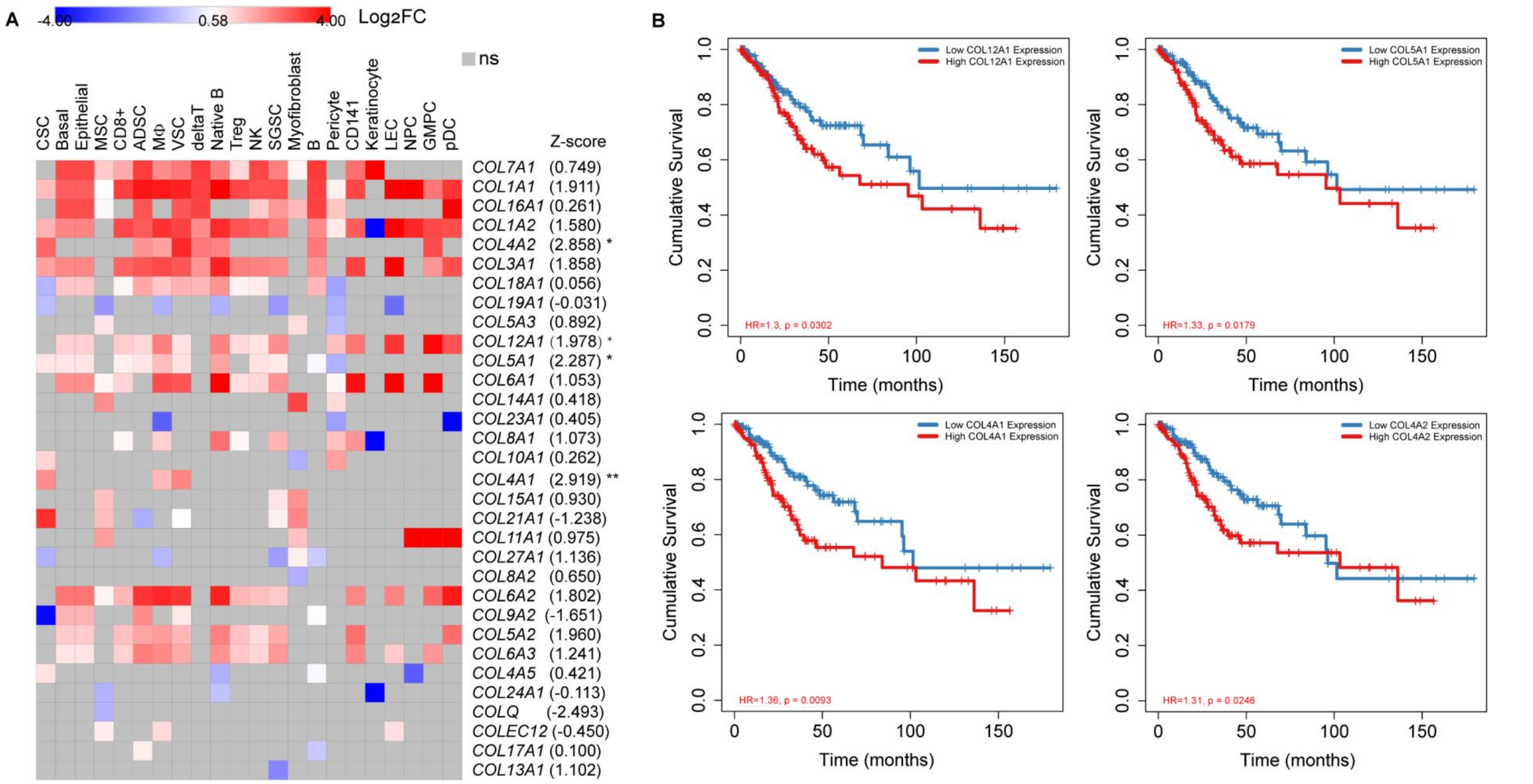

Fig. S9 The high relevance of collagens to CC patients and their survival outcomes. (**A**) The heatmap shows the expression (in Log_2FC value) of collagens significantly regulated in the CCII with respect to the CCI group. Z-score shows the correlation between the expression and the risk of CC derived from TCGA. *, *P*-value < 0.05; **, *P*-value < 0.01. (**B**) Association of COL12A, COL5A1, COL4A1 and COL4A2. with survival outcomes in CC patients.

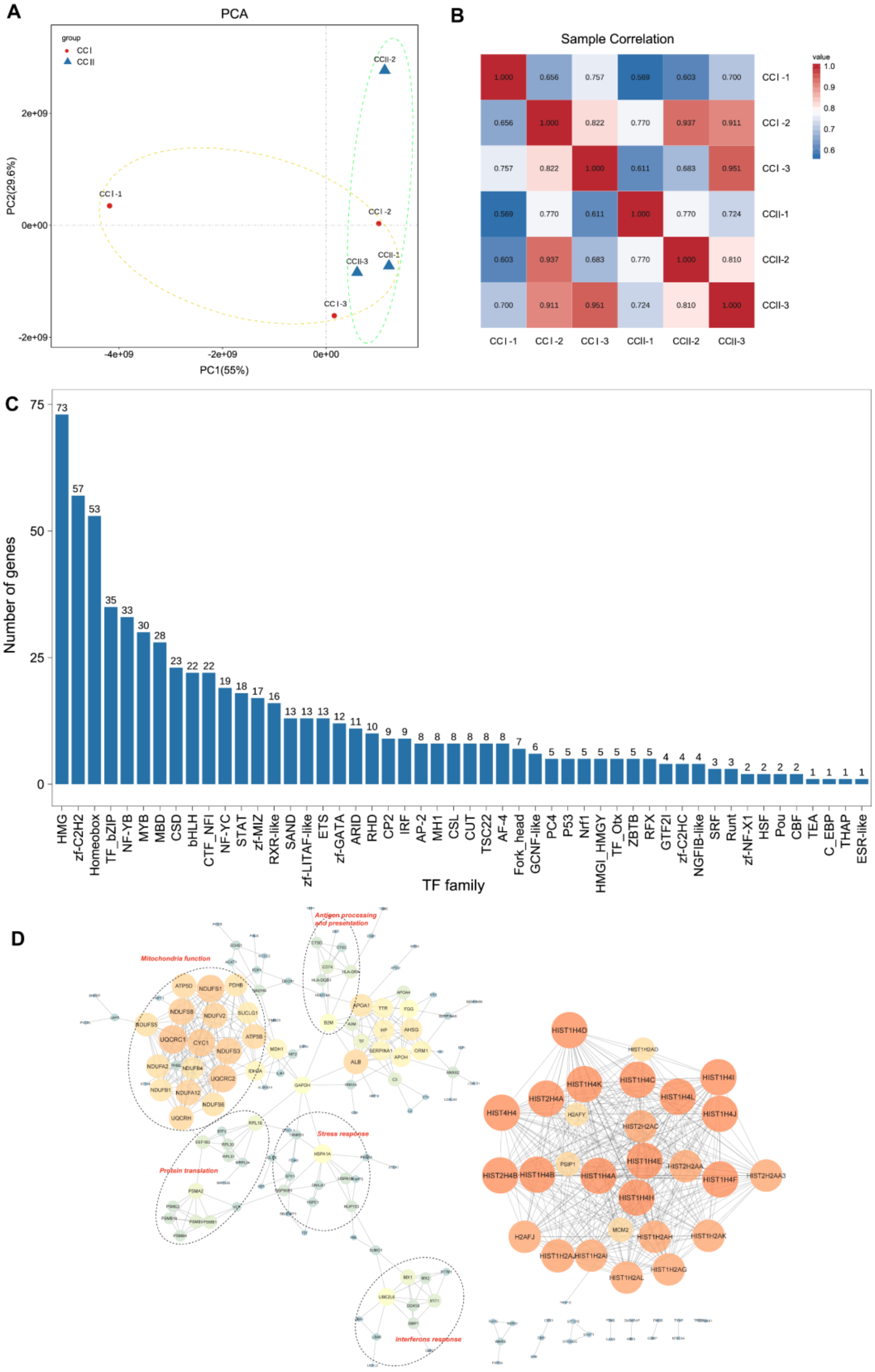

Fig. S10 (**A**) PCA analysis of the samples for proteomic analysis; (**B**) Correlation between the protein profiles of CCI and CCII samples; (**C**) Enrichment analysis of transcription factors (TFs); (D) protein-protein interactions between proteins significantly upregulated in the CCI group.

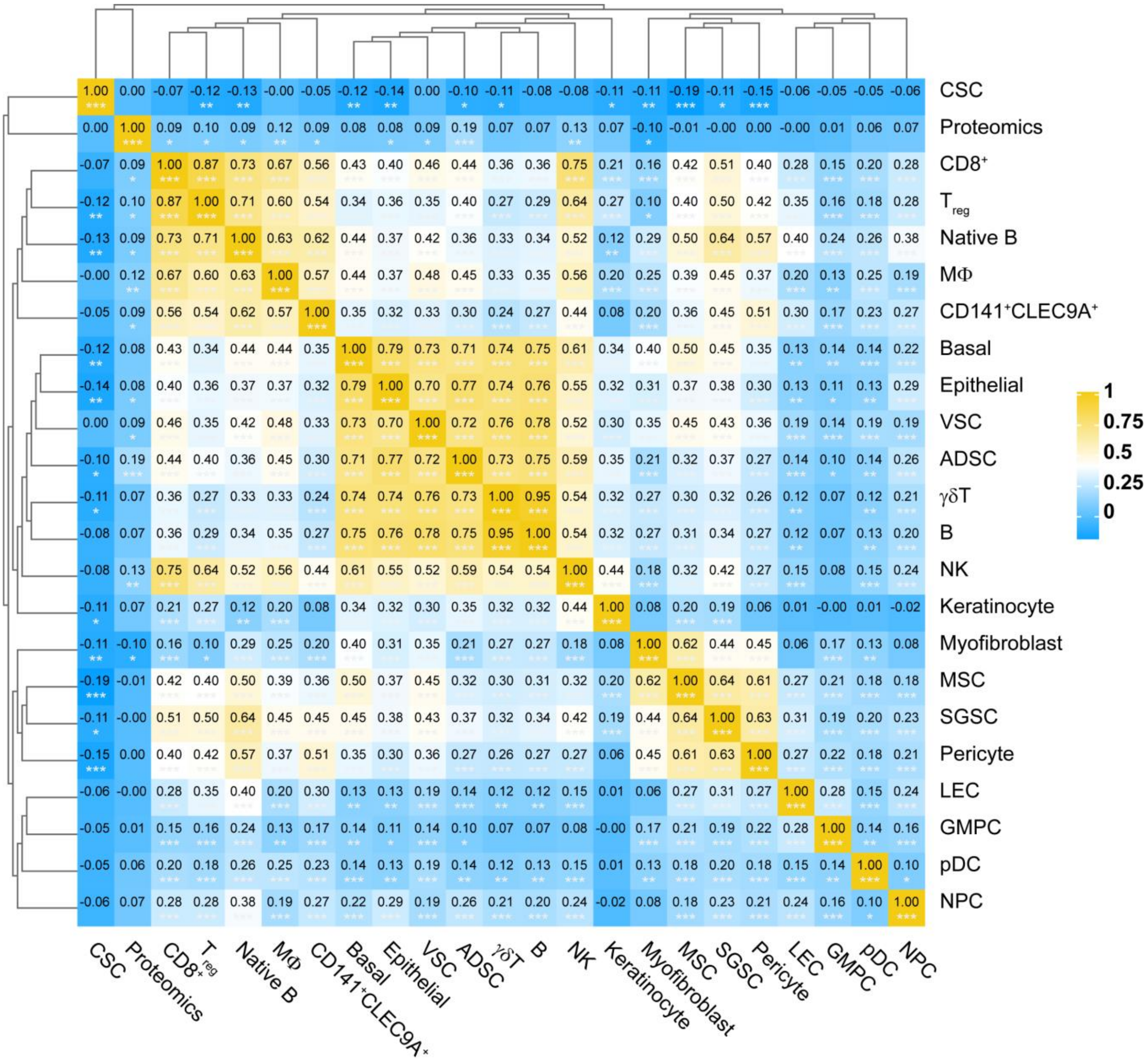

Fig. S11 Correlation analysis between all cell types and overall protein profile.

Supplementary tables

Table S1 Patient information and the populations of different cell types

Table S2 The normalised expression of top 5 marker genes of each cell cluster; all significantly upregulated genes of each cell cluster; the gene expression of the marker genes in all clusters. The annotations of these genes are shown.

Table S3 Comparative analysis of MΦs in CCI and CCII groups.

Table S4 Comparative analysis of MΦ subtypes and states in CCI and CCII groups.

Table S5 Comparative analysis of NK cells in CCI and CCII groups.

Table S6 Quantitative proteomic analysis results, including all identified proteins and supporting peptides.

Table S7 Comparative analysis of GO terms, KEGG pathways and TFs enriched by the DEGs between CCI and CCII groups.
